# Parallel evolutionary paths to produce more than one *Pseudomonas aeruginosa* biofilm phenotype

**DOI:** 10.1038/s41522-019-0113-6

**Published:** 2020-01-10

**Authors:** Janne G. Thöming, Jürgen Tomasch, Matthias Preusse, Michal Koska, Nora Grahl, Sarah Pohl, Sven D. Willger, Volkhard Kaever, Mathias Müsken, Susanne Häussler

**Affiliations:** 1 Institute for Molecular Bacteriology, TWINCORE, Centre for Experimental and Clinical Infection Research, Hannover, Germany; 2 Department of Clinical Microbiology, Copenhagen University Hospital – Rigshospitalet, Copenhagen, Denmark; 3 Department of Molecular Bacteriology, Helmholtz Centre for Infection Research, Braunschweig, Germany; 4 Research Core Unit Metabolomics and Institute of Pharmacology, Hannover Medical School, Hannover, Germany; 5 Central Facility for Microscopy, Helmholtz Centre for Infection Research, Braunschweig, Germany; 6 Cluster of Excellence RESIST (EXC 2155), Hannover Medical School, Hannover, Germany

**Keywords:** Biofilms, Next-generation sequencing

## Abstract

Studying parallel evolution of similar traits in independent within-species lineages provides an opportunity to address evolutionary predictability of molecular changes underlying adaptation. In this study, we monitored biofilm forming capabilities, motility, and virulence phenotypes of a plethora of phylogenetically diverse clinical isolates of the opportunistic pathogen *Pseudomonas aeruginosa*. We also recorded biofilm-specific and planktonic transcriptional responses. We found that *P. aeruginosa* isolates could be stratified based on the production of distinct organismal traits. Three major biofilm phenotypes, which shared motility and virulence phenotypes, were produced repeatedly in several isolates, indicating that the phenotypes evolved via parallel or convergent evolution. Of note, while we found a restricted general response to the biofilm environment, the individual groups of biofilm phenotypes reproduced biofilm transcriptional profiles that included the expression of well-known biofilm features, such as surface adhesive structures and extracellular matrix components. Our results provide insights into distinct ways to make a biofilm and indicate that genetic adaptations can modulate multiple pathways for biofilm development that are followed by several independent clinical isolates. Uncovering core regulatory pathways that drive biofilm-associated growth and tolerance towards environmental stressors promises to give clues to host and environmental interactions and could provide useful targets for new clinical interventions.

## Introduction

The ecological success of many opportunistic bacterial pathogens is based on their remarkable capability to adapt to, and survive in a broad range of diverse and challenging habitats.^1,2^ The ability to fine-tune the activity of a plethora of transcriptional regulators enables versatile lifestyles and flexible changes in bacterial behavior.^3–5^ This environment-driven transcriptional regulation increases the fitness of the individual bacterium.^6–9^ Additionally, specific genomic mutations help bacterial populations shape their behavior in distinct habitats.^10–12^

*Pseudomonas aeruginosa* is an environmental bacterium and opportunistic pathogen, which plays a dominant role as a causative agent of acute and chronic, often biofilm-associated, infections.^13–18^ Especially in cystic fibrosis (CF) patients, *P. aeruginosa* adopts a biofilm mode of growth, which contributes to high antibiotic tolerance and the recalcitrant nature of these infections.^19–24^ *P. aeruginosa* adapts to the hostile habitat of the chronically infected lung, producing a robust transcriptional response.^25–27^ Furthermore, ongoing inflammation generates very strong selective pressures, driving evolution and genotypic diversification of the infecting strain(s).^28–33^ Although the evolution of infecting bacteria present in advanced stages of CF infections has been extensively described,^34–36^ much less is known on how the observed genetic diversity is translated into better adapted phenotypes.^33,37^

In a previous study, we demonstrated that the variation width of the transcriptional profile (reaction norm) of one *P. aeruginosa* strain grown in different environmental conditions was much larger than the transcriptional variation among 151 genetically diverse clinical strains grown under standard laboratory conditions.^38^ However, various genotypes within a single species may also show differing reaction norms in different environments.^39^ Clinical *P. aeruginosa* isolates that have acquired a diverse array of adaptive mutations during an infection may produce more divergent transcriptional profiles when examined under infection-relevant conditions as opposed to standard laboratory conditions.

In this study, we recorded the transcriptional profiles of a diverse array of clinical *P. aeruginosa* isolates grown in planktonic and biofilm conditions. The latter is expected to more closely mimic conditions encountered during a chronic infection process. We demonstrate that while the transcriptional profiles elucidated in planktonic growth conditions were quite similar, more divergent transcriptional profiles were recorded when the isolates were grown in biofilm conditions. We also found that distinct groups of clinical isolates appear to follow parallel evolutionary paths and produce similar phenotypes. This convergence of organismal phenotypes was observed for multiple traits that included the formation of distinct biofilm structures characterized by specific transcriptional signatures, as well as virulence and motility phenotypes. Our results shed light on the enormous complexity that exists in the interrelationships between genetic and environmental factors in determining *P. aeruginosa* pathogenicity traits. As multiple genes interact with multiple environmental variables to produce a given phenotype, a holistic view of environmental and genetic effects on the bacterial phenotype will be critical to understand the intricate connection between genotypes and phenotypes.

## Results

### Biofilm phenotypes of clinical *P. aeruginosa* isolates fall into distinct clusters

**Fig. 1:**
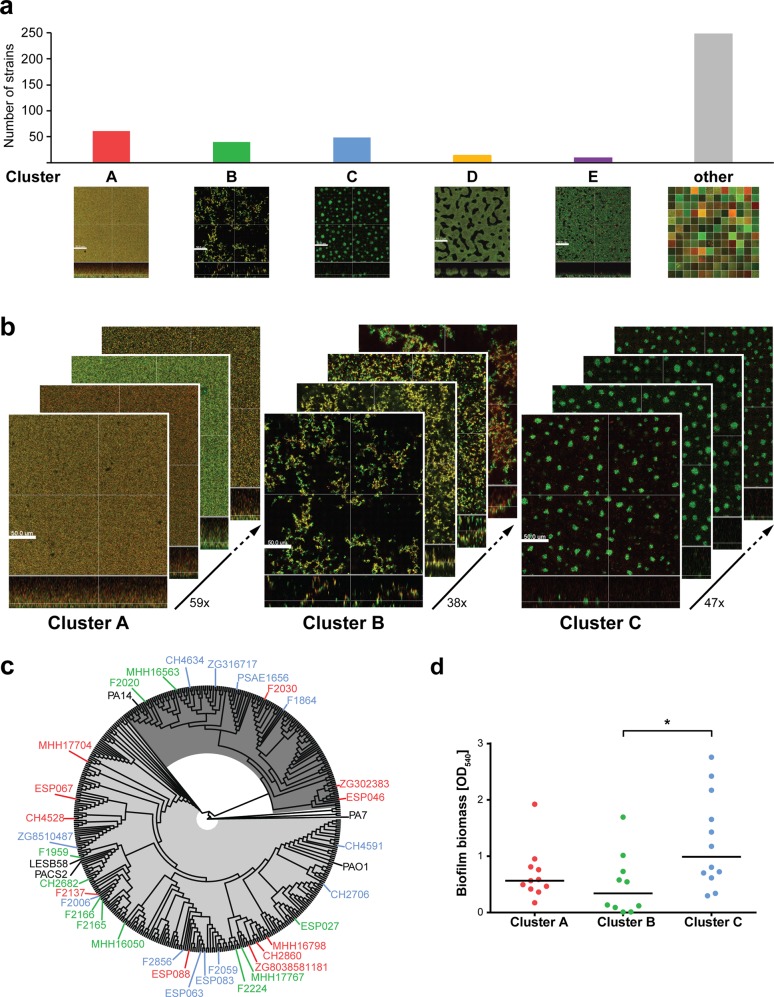
Biofilms of clinical *P. aeruginosa* isolates fall into three major clusters independent of their phylogenetic background. **a** Despite a large structural diversity in biofilms of 414 clinical isolates, groups of strains that share structural characteristics were identified by visual inspection of biofilm microscopy images. Biofilms were grown for 48 h in a microtiter plate-based in vitro biofilm assay; images were acquired using confocal laser scanning microscopy (CLSM) following live/dead staining. Living cells are displayed in green (Syto9); dead cells in red (propidum iodide: PI). 3D reconstructions were generated with the Imaris Software. The scale bar represents 50 µm. **b** Representative biofilm images of selected clinical isolates show exemplarily the structural characteristics of the three major biofilm clusters, of which each contains 59 strains (cluster A), 38 strains (cluster B), and 47 strains (cluster C), respectively. **c** The phylogenetic relationship of 33 representative clinical isolates is displayed in a phylogenetic tree based on 3524 genes that are present in the DNA sequences of 414 clinical isolates and 5 reference strains. The color code of the strain names represents the affiliation to a certain biofilm cluster: Red—cluster A; green—cluster B; blue—cluster C. Reference strains are displayed in black. The proportion of PAO1-like strains is highlighted in light gray; PA14-like strains are highlighted in dark gray. **d** Crystal violet quantification was performed for 33 representative clinical strains to assess air–liquid biofilm formation on a PVC surface after 24 h. Each datapoint represents one clinical isolate. Statistical significance was calculated using Tukey’s HSD (honest significant difference) following analysis of variance (ANOVA) and is displayed as *p < 0.05.

We have previously shown that clinical *P. aeruginosa* isolates from various patients and infectious sites exhibit very different in vitro biofilm structures.^40,41^ In this study, we recorded the biofilm phenotypes of 414 clinical *P. aeruginosa* isolates by the use of confocal laser scanning microscopy (CLSM) following live/dead staining. Clinical isolates exhibited a large diversity of biofilm phenotypes; however, on the basis of visual inspection of microscopy images, we identified clusters of strains that share common structural characteristics (Fig. 1a). We found that 138 (=33%) of the tested clinical isolates produced biofilm structures that could be grouped into three major clusters according to their biofilm phenotype: cluster A contained 59 strains, cluster B 38 strains and cluster C 47 strains. Figure 1b depicts exemplary CLSM images of *P. aeruginosa* isolates from each of these three biofilm clusters. The biofilms of clinical isolates belonging to cluster A showed overall weak fluorescence and were unstructured, or poorly structured. In contrast, cluster B isolates produced tall, highly structured and filamentous biofilms. Cluster C comprised isolates characterized by small microcolony-like aggregates in flat structures. In addition, minor clusters with <15 strains per group could be identified. Those consisted of isolates forming honeycomb-like biofilm structures (cluster D) or flat, coarsely cross-linked biofilms (cluster E) (Fig. 1a). Clinical isolates, which exhibited individual variations in the features represented in the major clusters or which exhibited very unique structural biofilm features, were categorized as unassigned (other).

Of note, the distinct phenotypes of the three major biofilm clusters were independent of the genetic background of the clinical isolates (Fig. 1c, Supplementary Fig. 1). Furthermore, the in vitro biofilm structures, as recorded by confocal microscopy, could not be deduced from crystal violet biofilm test assays. We observed large variations in the quantified surface-bound biomass within 10–12 isolates of each cluster (Fig. 1d). No significant differences between cluster A and B isolates were documented, while cluster C isolates exhibited an overall increased surface-bound biomass.

### Environment-dependent divergence of transcriptional profiles of clinical *P. aeruginosa* isolates

**Fig. 2:**
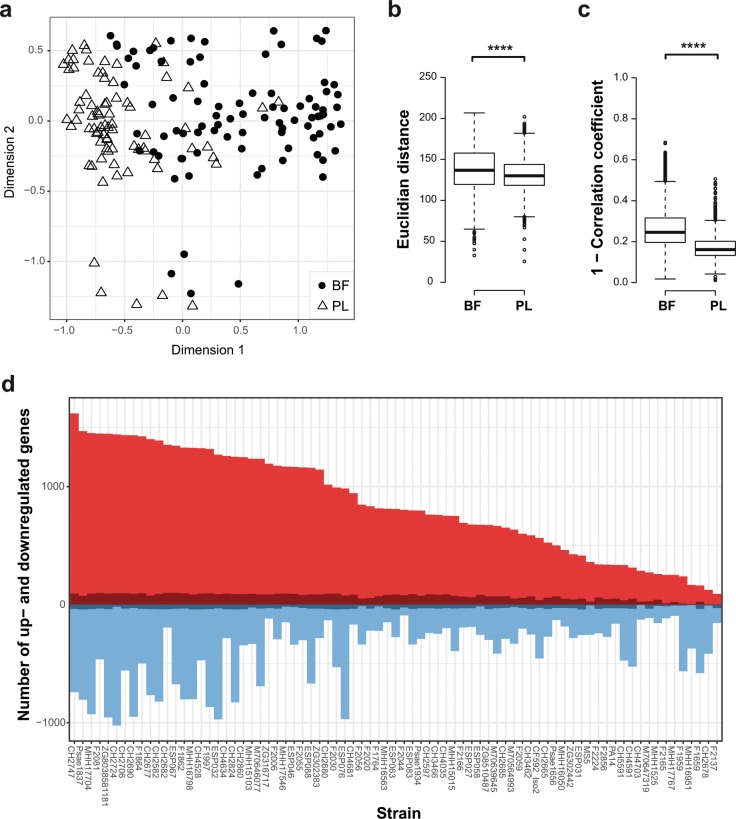
Transcriptional profiles recorded for biofilm-associated conditions exhibit a higher diversity than those recorded for planktonic conditions. **a** The multidimensional scaling plot (MDS) of transcriptional profiles of 77 clinical strains shows a higher divergence in biofilm conditions (BF; filled circles) as compared to planktonic conditions (PL; triangles). **b** Pairwise measurements of Euclidian distances and **c** the Pearson’s distance (Pearson correlation coefficient subtracted from 1 to describe the variance) between the samples of the two culture conditions (biofilm and planktonic) are depicted. Significance of the Wilcoxon’s rank sum-test is displayed as ****p < 0.0001. Boxplot elements are: center line—median; box limits—upper and lower quartiles; whiskers—1.5× interquartile range; points—outliers. **d** Clinical isolates show a broad range in the number of differentially expressed genes (biofilm versus planktonic). The number of core biofilm transcriptome genes present in each strain is displayed in dark red (upregulated in biofilms) and dark blue (downregulated in biofilms).

We next recorded the transcriptional profiles of a subgroup of 77 clinical *P. aeruginosa* isolates under planktonic (in a previous study^42^) and biofilm growth conditions (this study) (Fig. 2a). The selected 77 strains (Supplementary Table 1) represent the phylogenetic diversity of our entire collection of 414 strains and included strains of the three major biofilm clusters as well as strains exhibiting various other biofilm characteristics. As previously described,^38^ the transcriptional profiles of the majority of the clinical isolates were similar when grown under planktonic conditions. However, transcriptional profiles exhibited significantly higher diversity when biofilm-associated conditions were recorded. Measurements of the pairwise Euclidean distances revealed higher values among the biofilm samples than among the planktonic samples (Fig. 2b). We also found a higher pairwise Pearson’s distance among the biofilm-associated transcriptional profiles (Fig. 2c). Our results clearly show that the genomic make-up of the clinical isolates has a higher impact on the transcriptional profile under biofilm growth conditions than in planktonic cultures.

### The core biofilm transcriptome

To analyze whether the individual clinical strains share a common biofilm-specific transcriptional signature, we identified all genes that were differentially expressed between biofilm and planktonic growth conditions in each of the 77 clinical isolates (Fig. 2d). The number of differentially expressed genes (DEGs) between the two conditions ranged from 246 to 2465 per isolate (Fig. 2d). More than half of the identified genes (51%) were differentially regulated in 1–6 of the 77 clinical isolates (Supplementary Fig. 2).

We also recorded the genes that were differentially regulated between the biofilm and planktonic growth states if the respective transcriptional profiles of the individual 77 clinical isolates were considered as replicates. This core biofilm transcriptome consisted of overall 143 genes (Table 1, Supplementary Fig. 3). Although not all of these 143 genes were also differentially regulated at least two-fold in all individual isolates, more than 90% of the genes (130 of the 143 genes) were regulated in a minimum of 45 out of the 77 isolates (Supplementary Figs 2 and 3).

**Table 1:** The core biofilm transcriptome.

Upregulated genes								Downregulated genes			
Locus tag		Gene	Log 2 FC	Locus tag		Gene	Log 2 FC	Locus tag		Gene	Log 2 FC
PA14	PAO1			PA14	PAO1			PA14	PAO1		
PA14_06090	PA0466		2.9	PA14_38850	PA1983	*exaB*	6.8	PA14_04650	PA0355	*pfpI*	−3.2
PA14_06180	PA0472	*fiuI*	2.5	PA14_38860	PA1982	*exaA*	5.8	PA14_06650	PA0509	*nirN*	−3.0
PA14_07355	PA0565		3.6	PA14_38880	PA1981		5.7	PA14_06660	PA0510	*nirE*	−3.3
PA14_09980	PA4167	*dkgB*	2.7	PA14_38900	PA1980	*exaE*	2.9	PA14_06670	PA0511	*nirJ*	−3.5
PA14_10170	PA4159	*fepB*	3.2	PA14_38910	PA1979	*exaD*	3.0	PA14_06680	PA0512	*nirH*	−3.2
PA14_10200	PA4156		2.8	PA14_38920	—		4.1	PA14_06690	PA0513	*nirG*	−2.9
PA14_10240	PA4152	*acoC*	2.8	PA14_38970	PA1976		2.9	PA14_06700	PA0514	*nirL*	−3.3
PA14_10250	PA4151	*acoB*	3.0	PA14_38990	PA1975		3.1	PA14_06710	PA0515	*nirD*	−3.3
PA14_10260	PA4150	*acoA*	3.1	PA14_39000	PA1974		4.9	PA14_06720	PA0516	*nirF*	−3.1
PA14_10270	PA4149	*acoX*	3.5	PA14_39050	PA1971	*braZ*	2.5	PA14_06730	PA0517	*nirC*	−3.9
PA14_10280	PA4148		3.6	PA14_39260	PA1952		2.7	PA14_06740	PA0518	*nirM*	−4.8
PA14_10400	—		3.2	PA14_39590	PA1927	*metE*	4.3	PA14_06750	PA0519	*nirS*	−4.9
PA14_10410	—		2.9	PA14_39720	PA1918		2.6	PA14_06770	PA0520	*nirQ*	−3.3
PA14_11070	PA4085	*cupB2*	2.7	PA14_39750	PA1916		2.7	PA14_06810	PA0523	*norC*	−5.9
PA14_19270	PA3467		2.7	PA14_39860	PA1907		2.5	PA14_06830	PA0524	*norB*	−4.5
PA14_20010	PA3408	*hasR*	3.3	PA14_39980	PA1898	*qscR*	2.6	PA14_06840	PA0525	*norD*	−4.4
PA14_20020	PA3407	*hasAp*	5.1	PA14_40040	PA1893		2.7	PA14_06860	PA0526		−3.9
PA14_20030	PA3406	*hasD*	3.1	PA14_40050	PA1892		2.7	PA14_09610	PA4200		−2.5
PA14_20040	PA3405	*hasE*	3.0	PA14_40060	PA1891		3.0	PA14_09660	PA4198		−2.6
PA14_20050	PA3404	*hasF*	2.7	PA14_40270	PA1873		2.8	PA14_16640	PA3691		−2.9
PA14_21530	PA3287		3.1	PA14_40520	PA1855		2.7	PA14_20180	PA3394	*nosF*	−2.8
PA14_22320	PA3237		4.0	PA14_41510	PA1783	*nasA*	2.8	PA14_20190	PA3393	*nosD*	−2.8
PA14_27370	PA2840	*deaD*	2.4	PA14_41520	PA1782	*ppkB*	2.6	PA14_20200	PA3392	*nosZ*	−4.8
PA14_28100	PA2783		2.6	PA14_41530	PA1781	*nirB*	2.7	PA14_20230	PA3391	*nosR*	−4.3
PA14_28360	—		2.7	PA14_44190	PA1569		2.8	PA14_29640	PA2664	*fhp*	−3.8
PA14_28620	PA2746		3.2	PA14_44520	PA1541	*ydgF*	3.3	PA14_29660	PA2662		−2.6
PA14_29350	PA2688	*pfeA*	2.6	PA14_44530	PA1540	*ygdE*	2.8	PA14_40850	PA1831	*gpmA*	−2.4
PA14_29480	PA2678	*wzm*	2.7	PA14_46850	PA1347		2.8	PA14_50520	PA1074	*braC*	−2.5
PA14_32080	PA2518	*xylX*	3.3	PA14_47380	PA1302	*hxuC*	2.6	PA14_52660	PA0899	*aruB*	−2.3
PA14_32100	PA2517	*xylY*	2.9	PA14_47390	PA1301		3.0	PA14_52670	PA0898	*astD*	−2.2
PA14_33250	PA2427		4.4	PA14_47400	PA1300		2.8	PA14_52690	PA0897	*aruG*	−3.0
PA14_33260	PA2426	*pvdS*	3.0	PA14_49690	PA1137		3.8	PA14_52720	PA0895	*argD*	−2.4
PA14_33270	PA2425	*pvdG*	4.0	PA14_50050	PA1108		2.6	PA14_56370	PA4336		−2.9
PA14_33280	PA2424	*pvdL*	3.8	PA14_51460	PA0993	*cupC2*	3.2	PA14_56780	PA4366	*sodB*	−2.6
PA14_33500	PA2413	*pvdH*	3.0	PA14_52960	PA0874		2.7	PA14_57020	PA4386	*groES*	−2.7
PA14_33510	PA2412		3.5	PA14_53300	PA0848		3.4	PA14_60570	PA4578		−2.9
PA14_33520	PA2411		3.7	PA14_55500	PA0680	*hxcV*	2.6	PA14_61650	PA4661	*pagL*	−2.7
PA14_33600	PA2403	*fpvG*	3.1	PA14_56770	PA4365		2.5	PA14_64480	PA4876	*osmE*	−2.7
PA14_33610	PA2400		2.9	PA14_57990	PA4467		3.1	PA14_64520	PA4880		−2.9
PA14_33690	PA2397	*pvdE*	3.3	PA14_58000	PA4468	*sodM*	3.1	PA14_69770	PA5285		−2.6
PA14_33710	PA2395	*pvdO*	3.0	PA14_58010	PA4469		3.5				
PA14_33720	PA2394	*pvdN*	3.2	PA14_58030	PA4470	*fumC*	3.3				
PA14_33730	PA2393		3.6	PA14_58040	PA4471		3.9				
PA14_33810	PA2386	*pvdA*	4.5	PA14_60480	PA4570		4.1				
PA14_33820	PA2385	*pvdQ*	3.3	PA14_68430	PA5180	*fdhD*	2.9				
PA14_35430	PA2254	*pvcA*	2.7	PA14_68440	PA5181		2.5				
PA14_35460	PA2252		2.9	PA2958.1	PA2958.1	*rgsA*	3.7				
PA14_35980	—		2.9	PA3964-PA3965	PA3964-PA3965	P24	3.6				
PA14_37380	PA2097		2.4	PA4704.1	PA4704.1	*prrF1*	3.3				
PA14_38210	PA2034		2.6	PA4704.2	PA4704.2	*prrF2*	2.8				
PA14_38220	PA2033		2.6	PA4704-PA4705	PA4704-PA4705	*prrH*	3.1				
PA14_38310	PA2027		3.3								

The analysis of differentially expressed genes between transcriptional profiles of 77 clinical *P. aeruginosa* isolates recorded under static biofilm (BF) and planktonic (PL) growth conditions resulted in the identification of 143 genes. 103 genes were significantly upregulated; 40 genes were significantly downregulated (threshold: log 2 fold change [log 2 FC] ≤ −2 and ≥2 with a false discovery rate of FDR < 0.05). Additional information about the identified genes (e.g., FDR values, gene products, and pseudoCAP) are available in Supplementary Data 1

The core biofilm transcriptome consisted of 103 genes that were upregulated in biofilm growth conditions and 40 genes that were downregulated (Table 1). Among the 103 genes that were commonly upregulated in biofilms, we identified genes required for the biosynthesis of pyoverdine (*pvdAEGHLNOPQR, pvdS*),^43^ the small regulatory RNAs *prrF1*, *prrF2*, and *prrH*, and genes of the heme assimilation system (*hasAp*, *hasD*, *hasE*, *hasF*, *hasR*). Furthermore, genes encoding for a superoxide dismutase (*sodM*) and a fumarate hydratase (*fumC*) were upregulated, implying that cells within biofilms face iron-limiting conditions and oxidative stress. Moreover, genes involved in the central carbon catabolism and energy metabolism were identified among the upregulated genes in the core biofilm transcriptome. We found *acoABCX*, involved in the conversion of 2,3-butanediol into aldehydes,^44^ to be highly expressed under biofilm conditions as well as the ethanol oxidation genes (*exaABDE*), which catalyze the conversion of ethanol via acetaldehyde to acetate and subsequently to acetyl-CoA before its introduction into the glyoxylate cycle for further metabolism.^44^

Among the downregulated genes were genes involved in the denitrification pathway including genes of the *nir*, *nor*, and *nos* operons. Furthermore, genes of the arginine succinyltransferase (AST) pathway (*aruBG* encoding the AST; *astD* and *argD*) required for aerobic arginine catabolism were commonly downregulated.

### Convergence of biofilm transcriptional profiles

**Fig. 3:**
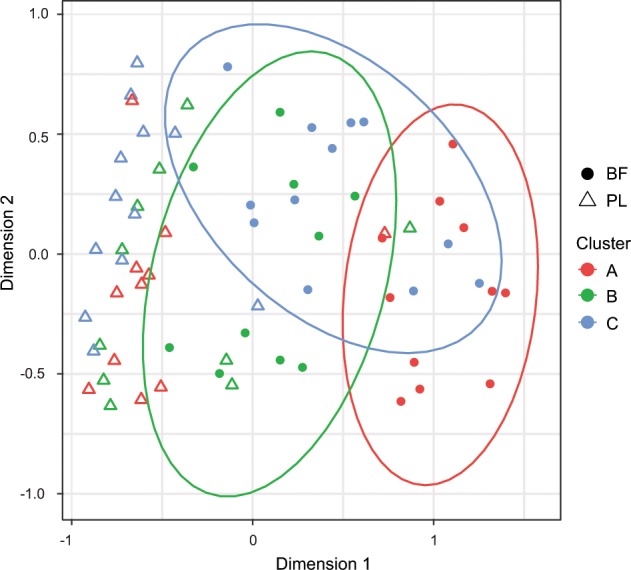
Biofilm clusters exhibit distinct transcriptional signatures in biofilm growth conditions. Biofilm (BF) but not planktonic (PL) transcriptional profiles show a grouping according to the biofilm structure. Ninety-five percent confidence intervals are displayed by ellipses. Each datapoint represents the transcriptional profile of one clinical isolate in a certain growth condition (BF: filled circles; PL: triangles). The color code represents the affiliation to a certain biofilm cluster: red—cluster A; green—cluster B; blue—cluster C.

We wondered whether the clinical strains that produced similar biofilm structures in our in vitro system (clusters A–C, Fig. 1b) also produce similar biofilm-associated transcriptional profiles. As demonstrated for the 77 clinical isolates (Fig. 2a), the transcriptional profiles of all 33 clinical isolates were very similar when grown under planktonic conditions, irrespective of their biofilm structure (Fig. 3). However, the biofilm-specific transcriptional profiles of the clinical isolates appeared to cluster according to their affiliation to a specific biofilm phenotype (Fig. 3, Supplementary Fig. 6).

### Genetic background rather than the maturation status of the biofilms impacts the biofilm transcriptional profile

The analysis of dynamic transcriptional profiles of a selection of six isolates (two from each of the three biofilm clusters) over time (12, 24, 36, and 48 h of biofilm growth) revealed robust gene expression patterns for each strain and biofilm phenotype. Clustering of the transcriptional profiles was impacted by the strain background and its affiliation to a specific biofilm cluster, rather than by the time at which the transcriptional profiles were recorded (Supplementary Fig. 4). Thus, the observed convergence of transcriptional biofilm signatures of the individual isolates within the clusters is shaped by the genomic make-up of the clinical isolates rather than by the maturation status of the biofilm.

### Cluster-specific biofilm transcriptional signatures

**Fig. 4:**
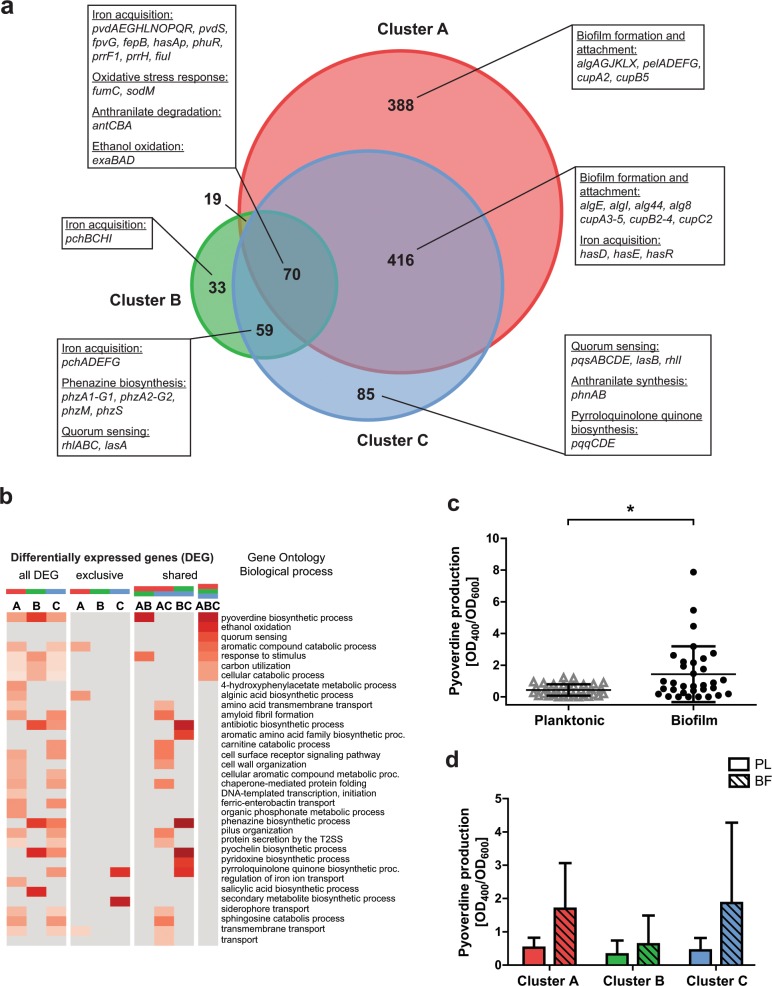
Upregulated genes in biofilm growth in comparison to planktonic growth. **a** The Venn diagram depicts commonly upregulated genes (70 genes) among all three biofilm clusters as well as cluster-specific regulated genes (A: 388; B: 33; C: 85 genes). **b** The GO term enrichment analysis of upregulated genes shows biological functions that are exclusively regulated in a certain biofilm cluster or shared by two or all three structural groups. The red color gradient represents the value of the enrichment factor. **c** A significant higher pyoverdine production was observed in biofilms (48 h) compared to planktonic cultures (24 h), as shown exemplarily for 33 clinical isolates. **d** Pyoverdine production in biofilm cultures was enhanced in all three biofilm clusters, independent of structural characteristics. Statistical significance was calculated with the Student’s *t* test and is displayed as *p < 0.05. Each datapoint represents one individual clinical isolate. Error bars represent the standard deviation.

Figure 4 and Supplementary Fig. 5 depict the number of differentially expressed genes (DEGs) under biofilm versus planktonic growth conditions (Fig. 4a and Supplementary Fig. 5a) and the enrichment of functional gene categories (Fig. 4b and Supplementary Fig. 5b) of regulated genes among the structural groups. In line with the finding that iron acquisition systems are a central part of the upregulated core biofilm transcriptome genes, all three biofilm clusters showed higher expression of genes involved in pyoverdine biosynthesis; an enhanced production of pyoverdine was measured in all isolates under biofilm growth conditions (Fig. 4c). Our results imply that the production of pyoverdine is a general biofilm trait that is independent of the biofilm structure (Fig. 4d). Additionally, we identified genes that were not part of the biofilm core, but are upregulated in all three biofilm clusters (Supplementary Fig. 6a), such as genes encoding the multicomponent anthranilate dioxygenase (*antABC*) (Fig. 4a).

We also identified cluster-specific biofilm gene expression patterns, indicating that there is a convergent evolution of distinct *P. aeruginosa* biofilm structures and transcriptional signatures. In the 11 cluster A isolates, which produced flat unstructured biofilms, 577 genes showed a specific expression profile in biofilms as compared to planktonic cultures (189 genes were downregulated, 388 genes were upregulated in biofilms) (Supplementary Data 1). We found a cluster A biofilm-specific upregulation of genes required for the production of extracellular polymeric substances. The functional analysis showed a significant enrichment for genes involved in the alginic acid biosynthetic process (*algAGJKLX*) (Fig. 4b). Further *alg* genes (*algE*, *algI*, *alg44*, and *alg8*) were upregulated in cluster A isolates; however, they were also found to be upregulated in cluster C isolates, which appear to share phenotypes consistent with both clusters A and B. Genes encoding the exopolysaccharide Pel (*pelADEFG*) and cup fimbrial genes (*cupA2* and *cupB2*) encoding adhesin-like proteins that are both involved in biofilm formation^45–47^ were found to be specifically upregulated in cluster A biofilms. Further, cup genes (c*upA3-5*, *cupB2-4*, *cupC2*) were upregulated in both cluster A and cluster C biofilms. Of note, under planktonic growth conditions, the expression of alginate, *pel* and *cup* fimbrial genes exhibited no significant difference between the isolates of the three different clusters (Supplementary Data 2).

Genes encoding the type IV pilus genes (e.g., *pilAOP*) showed significantly lower expression in cluster A during biofilm growth in comparison to planktonic growth. Furthermore, we found genes encoding components of the type III secretion system (T3SS; e.g., *pscD*, *exsC*, *pcrH*, *popB*) (Supplementary Fig. 5b). Interestingly, cluster A showed a specific downregulation of transcriptional regulators important for other virulence- and biofilm-related functions. For instance, the global regulator *mvaT* involved in controlling the expression of virulence factors, such as pyocyanin, swarming motility and protease,^48^ and biofilm formation,^49^ was lower expressed in cluster A biofilms. Moreover, the alternative sigma factor *sigX*, involved in the regulation of the T3SS, swarming motility, biofilm formation, and c-di-GMP signaling^50^ was expressed at lower levels. The master regulator *fleQ*, which not only regulates flagellar gene expression^51,52^ but also Pel biosynthesis in a c-di-GMP-dependent manner,^53^ was under-expressed in biofilm conditions in comparison to planktonic conditions.

Cluster B isolates, which produced tall, highly structured biofilms had a smaller number of regulated genes. Overall, 49 genes were specifically differentially regulated (16 downregulated and 33 upregulated) under biofilm growth conditions in cluster B. Genes that were exclusively upregulated in cluster B include those involved in pyochelin biosynthesis (*pchBCHI*). Further, pyochelin genes (*pchADEFG*) were upregulated in biofilms of clusters B and C. Moreover, quorum sensing-related genes (*rhlA*, *rhlB*, *rhlC*, and *lasA*) as part of global regulatory networks involved in environmental adaptation^54^ were upregulated in biofilms of clusters B and C.

Biofilms of clusters A and C had higher expression levels of *hasD*, *hasE*, and *hasR*, all encoding for components of the heme uptake system,^55^ in comparison to those found in cluster B. In both clusters B and C, *phz* genes (*phzA1-G1*, *phzA2-G2*, *phzM*, and *phzS*), required for the biosynthesis of pyocyanin,^56^ were upregulated during biofilm growth (Fig. 4b). Interestingly, analysis of transcriptional profiles under planktonic growth conditions revealed that cluster A already exhibited higher *phz* gene expression levels compared to clusters B and C (Supplementary Table 2).

106 genes were found to be exclusively regulated in cluster C biofilms (21 genes were downregulated, 85 genes were upregulated in biofilms). Phage-related genes (*gpFI*, *gpFII*, *gpI*, *gpW*) were specifically downregulated, while the *pqqCDE* operon required for the biosynthesis of pyrroloquinoline quinone, a redox cofactor for ethanol oxidation,^57^ was upregulated (Fig. 4b). Beaudoin et al.^58^ found that both *exaA* and *pqqC* are upregulated in *P. aeruginosa* PA14 biofilms and are linked to biofilm-specific tolerance. Furthermore, we observed a specific upregulation of *pqsABCDE* encoding 4-quinolone signal molecules in cluster C biofilms. Along this line, the expression of *phnAB*, encoding an anthranilate synthase, which is an important precursor of 4-quinolone production, was also found to be upregulated in cluster C isolates.

Notably, T3SS genes were found to have the highest expression in cluster C biofilms, whereas cluster A biofilms showed intermediate and cluster B biofilms the lowest expression levels. Under planktonic growth conditions, no significant differences in the expression of T3SS genes could be observed between the three clusters (Supplementary Table 3).

### Biofilm clustering correlates with other virulence-related *P. aeruginosa* phenotypes

**Fig. 5:**
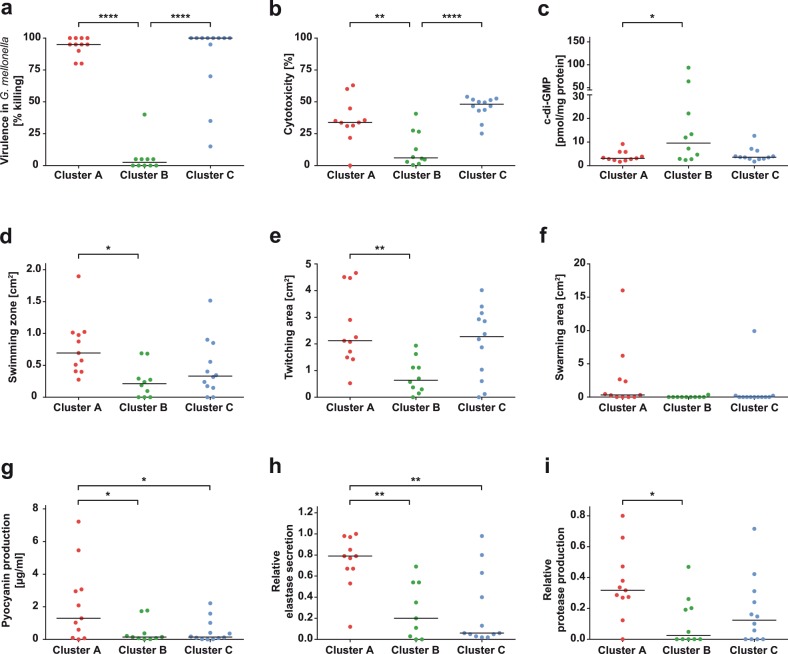
Biofilm cluster-specific isolates differ in their production of various virulence factors. **a** In vivo virulence using the *Galleria mellonella* model, **b** in vitro cytotoxicity on A549 epithelial cells, **c** intracellular second messenger c-di-GMP levels, **d** swimming motility, **e** twitching motility, **f** swarming motility, **g** pyocyanin production, **h** elastase secretion, and **i** protease production of selected isolates belonging to the three different biofilm clusters are depicted. Each dot represents one individual clinical isolate. Levels of statistical significance were calculated using Tukey’s HSD (honest significant difference) following analysis of variance (ANOVA) and are displayed as ****p < 0.0001, **p < 0.01, or *p < 0.05.

We screened the 33 aforementioned clinical isolates, as part of the three distinct biofilm clusters, for other phenotypes such as colony morphology, motility, and pathogenicity (Supplementary Table 4). We found that cluster A isolates produced colonies on blood agar plates with a strong hemolytic zone, while hemolysis was not as pronounced in colonies of *P. aeruginosa* isolates of the other two clusters (images available in the database bactome; https://bactome.helmholtz-hzi.de). Figure 5a depicts the in vivo virulence phenotype of cluster A, B, and C isolates as determined in the *Galleria mellonella* assay.^59^ In stark contrast to isolates of cluster A and C, isolates of cluster B were almost avirulent. An in vitro infection assay using human epithelial A549 cells showed a significant reduced cytotoxicity of cluster B isolates (Fig. 5b). Moreover, while cluster A isolates exhibited the highest motility, cluster B isolates showed the lowest motility in swimming, twitching, and swarming assays (Fig. 5d–f). Remarkably, the avirulent cluster B isolates, with an overall reduced motility, produced significantly higher levels of the intracellular second messenger c-di-GMP (Fig. 5c). Moreover, cluster A isolates secreted significantly higher levels of pyocyanin than the other two clusters (Fig. 5g), which is in agreement with the higher expression of *phz* genes (Supplementary Table 2). Cluster A strains also showed an enhanced protease secretion, as was found for proteolytic activity in general (Fig. 5i), and particularly elastase secretion (Fig. 5h).

## Discussion

The enormous success of environmental bacteria as human opportunistic pathogens is founded on their capability to efficiently adapt to the changing and challenging conditions within the human host.^1^ In general, there are two mechanisms that allow adaptation of a bacterial population to a changing environment: genetic diversification, which increases the ability of populations to adapt to new environmental conditions, and environment-driven transcriptional responses, which increase the fitness of the individual bacterium. It has become clear from many studies focusing on the various aspects of either of these two mechanisms that *P. aeruginosa* uses both strategies to adapt to host-specific niches.^25,26,32,34,35^ The complex interrelationships between genetic and environmental factors in determining bacterial traits, however, are largely unexplored and much remains to be learned about how genetic diversity is translated into differentially adapted phenotypes.

Similar to comparative genomics, comparative transcriptomics has the potential to uncover shared and unique features among multiple strains within the same species or among isolates that produce a specific organismal trait despite variation in environmental conditions.^60^ The detection of common responses of bacterial strains to a biofilm growth environment has been a focus of previous research.^61–64^ Uncovering core regulatory pathways that drive biofilm-associated tolerance towards environmental stressors promise to give clues to host and environmental interactions and could provide useful targets for new clinical interventions.

Previous studies described that different environmental conditions promote different biofilm-specific transcriptional profiles, and only a small number of common biofilm-specific transcripts could be detected.^65,66^ We found that a diverse array of clinical isolates shares a restricted core biofilm transcriptional profile, even if the isolates were cultivated under the same biofilm-promoting conditions. Interestingly, expression of previously well-described biofilm-associated genes, such as those involved in surface attachment and the production of biofilm matrix components,^67–69^ were not among the strict core biofilm genes. Instead, the core biofilm transcriptional profile was dominated by the expression of genes involved in stress responses and adaptation to oxygen and iron-limiting growth conditions.

Closer inspection revealed that clinical *P. aeruginosa* isolates can be stratified based on the production of specific biofilm structures in vitro. The individual biofilm phenotypes evolved via parallel or convergent evolution and were produced repeatedly in several clinical isolates. Interestingly, the individual groups of biofilm phenotypes produced biofilm transcriptional profiles that included the expression of surface adhesive structures and extracellular matrix components, indicating that there is more than one way to make a biofilm^70,71^ even under identical environmental conditions.

The independent evolution of specific biofilm phenotypic traits in non-related clinical isolates indicates adaptation to similar environments. Of note, we found that the biofilm-specific grouping of the clinical isolates was also reflected in convergent motility and virulence phenotypes. Our data thus show that the groups of clinical isolates have undergone different paths of convergent evolution to produce a complex phenotype that goes beyond the production of a distinct biofilm phenotype. In this context, our observation that the variation of the transcriptional profiles of our clinical isolates was larger under biofilm growth conditions than under planktonic conditions is interesting. The conserved lower variation width of the transcriptional profile of the evolved strains under planktonic growth conditions could promote maintenance of genetic diversity within the population, as the pressure to change decreases.

## Methods

### Strains, media, and growth conditions

To determine biofilm structures in a high content screen, we used our strain collection of 414 well-characterized clinical *P. aeruginosa* isolates, for which in vivo virulence data in the *Galleria mellonella* infection model^59^ and images of the colony morphology (including hemolysis on Columbia agar)^72^ were available. Furthermore, transcriptional profiles under planktonic conditions have been recorded for all 414 strains in the frame of a previous study.^42^ In this study, we performed transcriptional profiling under biofilm growth conditions for a subset of 77 strains (Supplementary Table 1). All clinical strains used in this study were collected in clinical microbiology laboratories, in private practice laboratories, or were provided by strain collection curators. Strains originate from sampling sites across Germany, Spain, Hungary, and Romania. Unless otherwise stated, all experiments were performed at 37 °C in standard lysogeny broth (LB) in order to accommodate growth and to enable comparable growth rates for clinical strains originating from diverse infection sites. Planktonic cultures were incubated in an orbital shaker (180 r.p.m.); biofilms were cultivated under static conditions in a humid atmosphere.

### Biofilm phenotype assay

A collection of 414 clinical *P. aeruginosa* isolates, which have previously been sampled from several clinical microbiology laboratories across Europe,^42,59,72^ was screened for biofilm phenotypes through the use of a high content static microtiter plate assay combined with automated CLSM.^73^ In brief, bacteria were grown overnight in LB at 37 °C and 100 µl of bacterial suspension with an adjusted OD_600_ of 0.002 were added to the wells of a sterile half-area 96-well µClear microtiter plate (Greiner Bio-One, Austria). The microtiter plate was sealed with an air-permeable BREATHseal cover foil (Greiner Bio-One) and incubated for 48 h in humid atmosphere at 37 °C. After 24 h, bacteria were stained by carefully adding 60 µl of a solution containing the fluorescent dyes Syto9 and propidium iodide (final concentrations of 2.1 and 12.5 µM, respectively) from the LIVE/DEAD^®^ *Bac*Light™ Bacterial Viability Kit (Molecular Probes, Life Technologies, CA, USA). Z-stacks of 48-h-old biofilms with a total height of 60 µm (20 focal planes; z-step size 3 µm) in the center of each well were acquired by using an automated confocal laser scanning microscope (SP8 System, Leica, Germany), including the matrix screener tool and equipped with an HC PL APO ×40/1.10 W motCORR CS2 water immersion objective. Imaris 7.6 (Bitplane, UK) was used for 3D reconstructions of biofilm structures. The biofilm phenotype was recorded for all clinical strains in four replicates. Major biofilm clusters with distinct structural characteristics were identified on the basis of microscopy images and clinical isolates were manually categorized by visual inspection. Additional minor biofilm clusters were not further described. For an accurate grouping, only strains that clearly reflected the respective structural properties of a certain biofilm cluster were considered for further investigation. Strains that did not fit the described selection criteria remained unassigned.

### Crystal violet biofilm formation assay

Selected clinical isolates were subjected to a crystal violet biofilm formation assay with slight modifications according to a previously published method.^74^ Briefly, overnight cultures were adjusted to an OD_600_ of 0.02 and 100 µl of the inoculum were added to each well of a flexible, non-treated U-bottom PVC 96-well plate (Corning Inc., NY, USA). Plates were sealed with an air-permeable membrane and incubated at 37 °C in humid atmosphere for 24 h. Planktonic bacteria were removed and wells were washed with water prior to the addition of 150 µl of the crystal violet staining solution (0.1% w/v in water). After 30 min incubation, the staining solution was removed and wells were again washed and air-dried. For de-staining, 200 µl of 96% ethanol was added to each well and the plate was incubated for 30 min at room temperature (RT). 125 μl of the solution was transferred to a fresh 96-well flat bottom plate before absorbance was measured at 540 nm. Each clinical isolate was tested in two independent experiments with eight technical replicates each time.

### RNA sequencing and bioinformatic analyses

We selected a subset of 77 clinical *P. aeruginosa* strains for transcriptional profiling of bacteria grown under both biofilm and planktonic conditions. Transcriptional profiles under planktonic conditions have been recorded in a previous study.^42^ Briefly, planktonic bacteria were cultivated to early stationary phase (OD_600_ = 2) in 10 ml LB under shaking conditions (37 °C, 180 r.p.m.). Three independent cultures were pooled and an equal volume of RNAprotect (Qiagen, The Netherlands) was added prior to cell harvest. Biofilm cultures were grown as described above. Dynamic transcriptional profiles were recorded over time. For this purpose, biofilm-grown cells were not only harvested at 48 h but also at 12, 24, and 36 h. To examine structural characteristics, all biofilms were evaluated by CLSM prior to harvest. At least ten wells of two biological replicates were pooled to obtain one transcriptional profile per strain and time point. Biofilm suspensions were mixed with an equal volume of RNAlater (Qiagen) and centrifuged. For RNA extraction, bacterial pellets were stored overnight at −80 °C and the RNeasy© Mini Kit (Qiagen) following QIAshredder™ columns (Qiagen) was used according to the manufacturer’s instruction. DNase treatment (DNA-free™ Kit DNase Treatment & Removal, Ambion, Life Technologies) was applied to remove remaining DNA. RNA samples were quality-checked by the use of the RNA Nano Kit with an Agilent Bioanalyzer 2100 (Agilent Technologies, CA, USA). To remove ribosomal RNA, the Ribo-Zero Bacteria Kit (Illumina, CA, USA) was used and cDNA libraries were generated using ScriptSeq™ v2 Kit (Illumina). Samples were sequenced in single-end mode on an Illumina HiSeq 2500 device (1 × 50 bp reads) or paired-end mode on a Novaseq (2 × 50 bp).

Reads were quality controlled and adapter clipped using fastq-mcf from the ea-utils package^75^ and mapped to the genome of the reference genome UCBPP-PA14 (NC_008463.1, available for download from the *Pseudomonas* Genome database: http://v2.pseudomonas.com) with bowtie2^76^ using default parameters for single- or paired-end reads. The resulting sam-files were converted to indexed binary format and pile-up format using SAMtools.^77^ The program *featureCounts*^78^ was used to count the reads mapping to genes.

Read counts were used as the basis for further analyses. Differential gene expression analysis between biofilm and planktonic transcriptional profiles was performed with the R package edgeR (v.3.20.1).^79^ Normalization factors to scale the raw library sizes were calculated using the weighted trimmed mean of *M* values method. Multidimensional scaling plots were generated using the plotMDS function of edgeR taking all genes into account. Normalized read counts were used to calculate the Euclidean and Pearson’s distance (1 − Pearson’s correlation) between samples. Therefore, basic R functions dist and cor were used, respectively.

Differential gene expression (biofilm vs. planktonic growth) was calculated for all 77 clinical isolates to identify the biofilm core transcriptome. For this purpose, the 77 strains were regarded as replicates. For the analysis of DEGs in biofilm clusters, 10 to 12 strains per each structural group were regarded as replicates. A fold change of 4 (log 2 fold change [log 2 FC] 2) was used as a threshold with a false discovery rate (FDR) of <0.05. The identified genes were used as the input for a Venn diagram to identify the overlap of regulated genes between the three biofilm groups. Area-proportional Venn diagrams were adapted from the online tool BioVenn.^80^ Overlapping DEGs and the output of the Venn diagram were used for an enrichment analysis. Functional enrichment of Gene Ontology terms^81,82^ was performed using the hypergeometric test (R function phyper, adjusted *p* value (FDR) < 0.05). For the analysis of DEGs between structure-related isolates (cluster-wise pairwise comparison), the threshold was set to an FC of 2.83 (log 2 FC 1.5) with an FDR < 0.05.

Correlation between biofilm clusters and phenotypes of the isolates was assessed by analysis of variance, followed by Tukey’s post hoc test. Both were performed using basic functions provided by the stats package in the R statistical environment.^83^

A phylogenetic tree was created based on whole-genome sequencing data, which are available for all 414 of our clinical *P. aeruginosa* isolates^42^ and five additional reference strains (UCBPP-PA14, PAO1, PA7, LESB58, PACS2). Sequences from 3524 genes that were present in all strains were used. Gene sequences for the isolates were generated from DNA sequencing reads using the *mpileup* option of the SAMtools package.^84^ Phylogenetic distances were calculated based on 17-mers to create an alignment-free k-mer tree.^85^ These distances were then used to visualize the phylogeny using the neighbor-joining tree estimation method from the R package ape and the package ggtree.^86,87^

### Quantification of cyclic diguanylate

Intracellular cyclic diguanylate (c-di-GMP) was extracted, and quantified by high-performance liquid chromatography-coupled tandem mass spectrometry according to a published protocol.^50,88^ In brief, bacterial cultures were grown for 24 h shaking at 37 °C and 5 ml of the bacterial suspensions were harvested. Isotope-labeled [^13^C^15^N]-c-di-GMP was used as an internal standard during methanol:acetonitrile:water (2:2:1) extraction. Intracellular c-di-GMP was determined for each clinical isolate in three independent experiments and the resulting concentration is given as pmol c-di-GMP/mg protein. Roti Nanoquant solution (Carl Roth, Germany) was used according to the manufacturer’s instructions to determine protein concentrations in a Bradford assay.

### In vitro cytotoxicity

In vitro infection assays were performed in accordance with published protocols.^50,89,90^ Briefly, A549 cells from a human alveolar adenocarcinoma cell line (ACC 107) were maintained in Dulbecco’s modified Eagle medium (DMEM) (Gibco, Life Technologies) supplemented with 2 mM l-glutamine (Thermo Fisher Scientific, MA, USA), 1× non-essential amino acids (Gibco), 100 U/ml penicillin–streptomycin (Gibco), and 10% fetal calf serum (Sigma-Aldrich, MO, USA). For the infection assay, A549 cells were grown in 24-well plates at 37 °C and 5% CO_2_ to 90% confluence. Prior to bacterial infection, epithelial cells were washed once with PBS. Bacterial inocula in DMEM were prepared from planktonic cultures in early stationary phase (3 to 3.5 h of growth) with an adjusted bacterial cell number according to a multiplicity of infection (MOI) of 10. To facilitate contact between bacteria and eukaryotic cells, plates were centrifuged for 5 min at 500 × *g* and incubated for 24 h at 37 °C. Supernatants were collected and centrifuged to pellet out cell debris and bacterial cells. The supernatant was used for the Pierce lactate dehydrogenase assay according to the manufacturer’s instructions (Thermo Fisher Scientific). As a positive control, one well was treated with lysis buffer 45 min prior to the collection of the supernatant, resulting in maximum cytotoxicity (cytomax). Cytotoxicity for clinical strains is given as percentage of cytomax.

### Motility assays

Motility experiments were carried out according to previously published protocols^91^ with slight modifications. For a screening attempt, swimming assays were carried out in 12-well plates (Nunc, Denmark) with 2.5 ml of BM2 glucose agar (62 mM potassium phosphate buffer [pH = 7], 7 mM (NH_4_)_2_SO_4_, 2 mM MgSO_4_, 10 µM FeSO_4_, 0.4% [w/v] glucose; containing 0.3% agar) per well. For swarming, BM2 medium without (NH_4_)_2_SO_4_ was supplemented with 0.1% casamino acids and 0.5% agar. Liquid cultures inoculated from overnight cultures were incubated until an OD_600_ of ~1.5 to 2.5 (log phase) was reached. Subsequently, all cultures were adjusted to an OD_600_ of 1. To evaluate swimming ability, 1 µl of the adjusted suspension was carefully added on top of the agar; for swarming 0.5 µl of the cellular suspension was pipetted into the agar. Plates were incubated for 15 h at 30 °C in a humid atmosphere prior to evaluation. For twitching assays, a hole was punched into LB agar plates solidified with 1.5% agar and pelleted bacteria from 1 ml of culture were carefully added onto the bottom of the petri dish. After 48 h of static incubation at 37 °C (humid atmosphere), the agar was removed and the twitching zone on the plastic surface of the petri dish was measured. For all motility experiments, two independent experiments were performed, including the lab strain PA14 as a reference. For evaluation, pictures were taken and the motility area for each clinical isolate was measured with ImageJ and adjusted to the respective PA14 control to account for inter-experimental variations.

### Pyocyanin production

Pyocyanin production was analyzed as described elsewhere.^92,93^ Briefly, cultures were grown for 24 h shaking before harvesting 5 ml bacterial suspension. After centrifugation, pyocyanin was extracted by the addition of an equal volume of chloroform. Three milliliters of the organic phase was mixed with 1 ml 0.2 M hydrochloric acid (HCl) and absorbance was determined at 520 nm for the aqueous phase. Pyocyanin concentrations [µg/ml supernatant] were calculated by multiplication with the correction factor 17.072.

### Pyoverdine production

To investigate pyoverdine production, clinical isolates were cultivated in iron-restricted succinate medium (6 g K_2_HPO_4_, 3 g KH_2_PO_4_, 4.2 g (NH_4_)_2_HPO_4_, 0.2 g MgSO_4_, 4 g succinic acid, 1.1 g NaOH; filled up to 1 l; pH = 7) as previously described.^94,95^ Clinical isolates were cultivated under both planktonic (16 h shaking) and biofilm conditions (48 h statically). Pyoverdine-specific absorbance was measured at 400 nm and normalized to bacterial growth (OD_600_).

### Proteolytic activity

To assess proteolytic activity of clinical strains, a protocol modified from Casilag et al.^90^ was used. The assay was performed with cation-adjusted Müller–Hinton (MH) broth (Fluka analytical, Thermo Fisher Scientific) containing 10% (v/v) of commercially available homogenized, ultra-heated milk (fat content < 0.3%), solidified with 1.5% agar. Overnight cultures were adjusted to an OD_600_ of 0.025 and 5 µl of the bacterial suspensions were spotted on top of the agar plates. Plates were incubated for 24 h at 37 °C and the clearing zones, which indicate proteolytic activity, were measured in axial and horizontal direction. Relative protease activity was calculated as follows:

1 $${\mathrm{Rel}}.{\mathrm{protease}}\,{\mathrm{activity}} = \frac{{{\mathrm{diameter}}\left( {{\mathrm{halo}}} \right) - {\mathrm{diameter}}({\mathrm{colony}})}}{{{\mathrm{diameter}}\,({\mathrm{colony}})}}.$$

### Elastase secretion

The elastolytic activity of proteases secreted by the clinical isolates was tested in an Elastin Congo Red (ECR) assay.^96^ Briefly, bacteria were cultivated in either planktonic or static conditions to promote biofilm formation. The standard biofilm assay was scaled up (15 times the volume) to 24-well plates (Nunc). Cells were harvested after 24 h (PL) or 48 h (BF), respectively, and 100 µl of the supernatant was mixed with 900 µl ECR buffer (100 mM Tris [pH 7.5], 1 mM CaCl_2_, supplemented with 22.5 mg/ml ECR [Sigma-Aldrich]). The suspension was incubated for 3 h at 37 °C and 900 r.p.m. After centrifugation, the absorbance of the supernatant was determined at 495 nm. Elastase secretion is indicated relative to the maximum absorbance.

### Oxidative stress response

To investigate the tolerance of clinical strains to H_2_O_2_, a disk diffusion assay was applied.^97,98^ In brief, bacteria were grown overnight and adjusted to an OD_600_ of 3. 150 µl of the bacterial suspension were mixed with 3 ml of cooled down LB soft agar (0.6% w/v) and poured onto the surface of LB solidified with 1.5% agar. Sterile filter paper disks were placed on the overlaying soft agar and 8 µl of 30% H_2_O_2_ (Carl Roth) was added. Plates were incubated for 16 h at 37 °C, and the diameter of the inhibition zone was measured in both the vertical and horizontal direction. H_2_O_2_ sensitivity is depicted as the mean of three technical triplicates.

### Reporting summary

Further information on research design is available in the Nature Research Reporting Summary linked to this article.

## Supplementary information

Supplementary Information

Supplementary Data 1

Supplementary Data 2

Reporting Summary

## Data Availability

Transcriptome data have been deposited to NCBI Gene Expression Omnibus (https://www.ncbi.nlm.nih.gov/geo/) under the accession numbers GSE134231 (biofilm transcriptomes) and GSE123544 (planktonic transcriptomes). DNA-seq data are available in the Sequence Read Archive (https://www.ncbi.nlm.nih.gov/sra) under the reference number PRJNA526797.

## Code availability

Scripts for RNA-seq data processing and analysis as well as for phylogenetic analysis are available from the authors upon request.
