# Supplementary material for: Parallel evolutionary paths to produce more than one Pseudomonas aeruginosa biofilm phenotype
Source: NPJ Biofilms Microbiomes. 2020 Jan 10;6:2. doi: 10.1038/s41522-019-0113-6 (PMC6954232; doi:10.1038/s41522-019-0113-6)
Supplement: Supplementary file 1 — Supplementary Information [file 41522_2019_113_MOESM1_ESM.pdf]

Supplementary Information

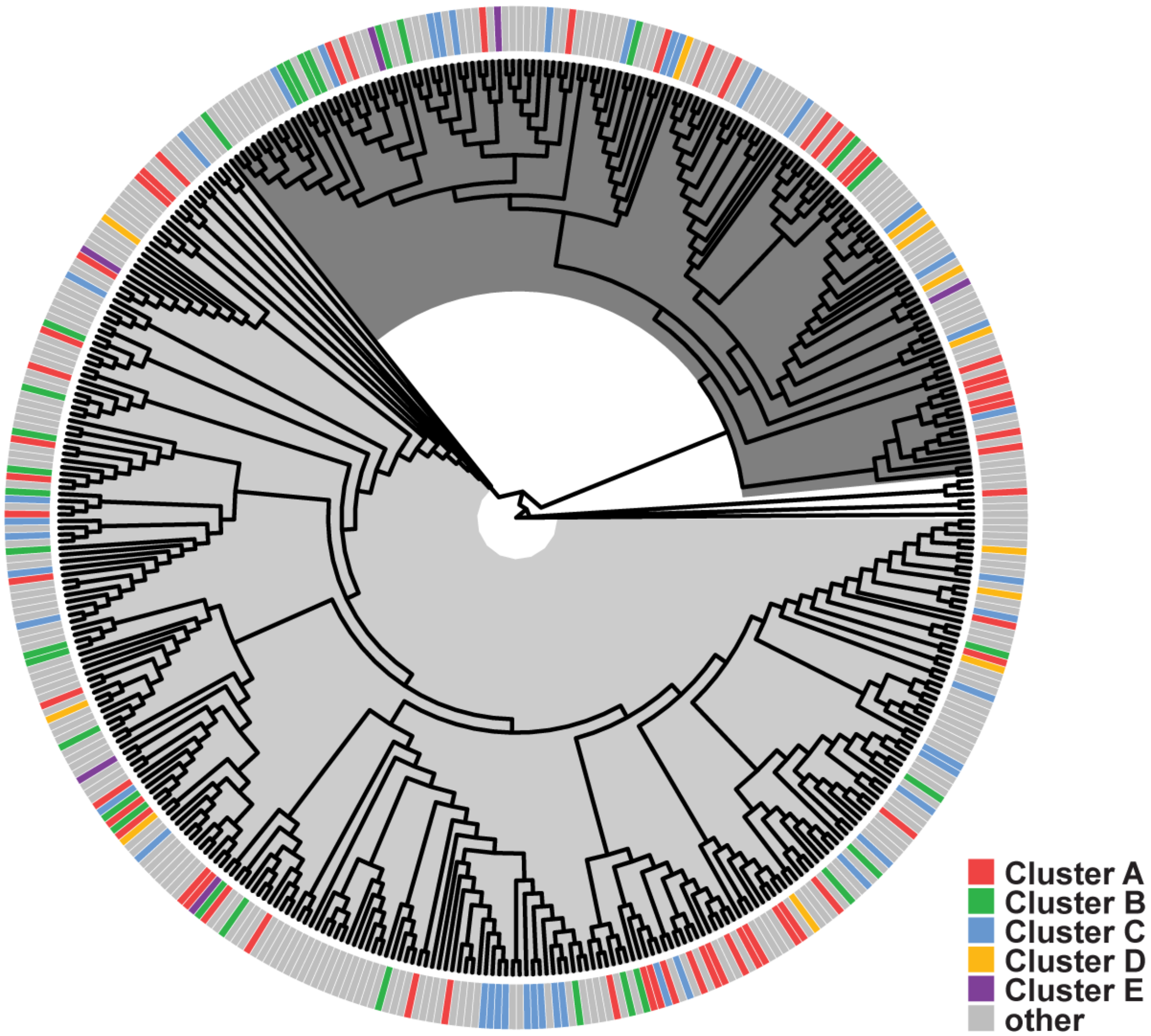

Supplementary Figure 1: The biofilm phenotype is independent of the genetic background and structurally related strains are distributed across the entire phylogenetic tree of 414 clinical *P. aeruginosa* isolates.

The phylogenetic tree is based on 3,524 genes that are present in the DNA sequences of 414 clinical isolates and 5 reference strains (PA14, PAO1, PA7, LESB58, PACS2). The proportion of PAO1-like strains is highlighted in light gray; PA14-like strains are highlighted in dark gray. The color code represents the assignment to a specific biofilm cluster: red – cluster A; green – cluster B; blue – cluster C; yellow – cluster D; purple – cluster E; gray – unassigned/others.

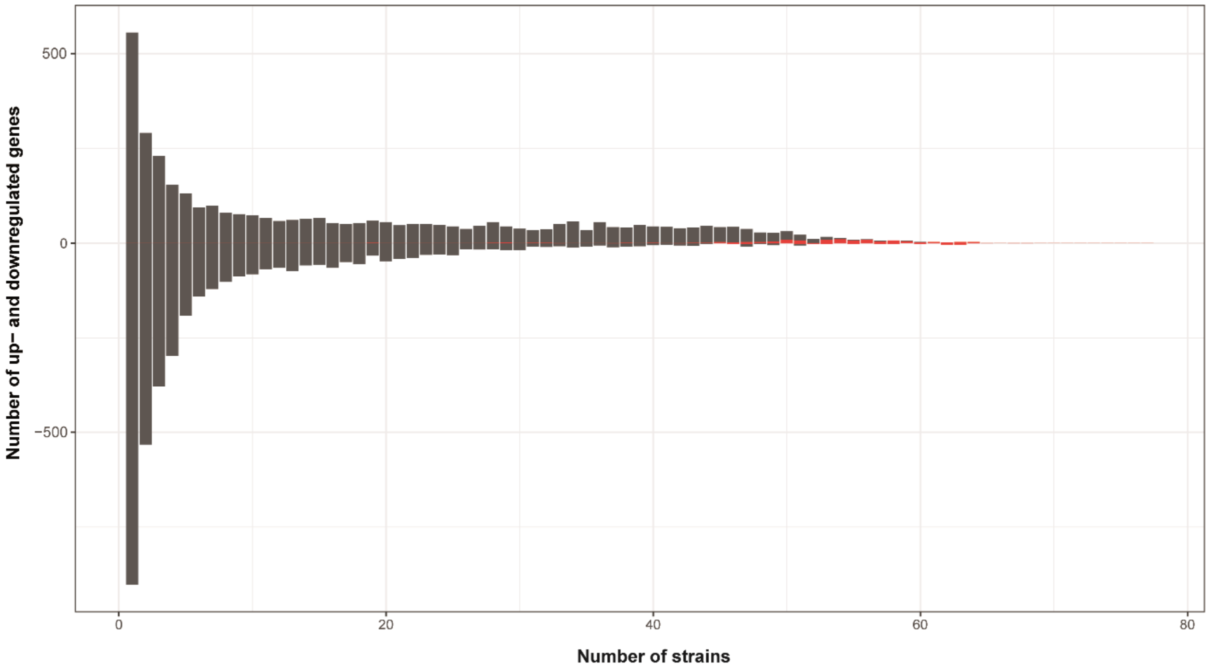

Supplementary Figure 2: Numbers of differentially expressed genes (biofilm vs. planktonic) that are commonly regulated among the tested strains.

A large number of genes are differentially regulated in only a few clinical isolates (≤ 6 strains), whereas genes of the biofilm core transcriptome (highlighted in red) are mostly shared among a large number of clinical isolates (≥ 45 strains).

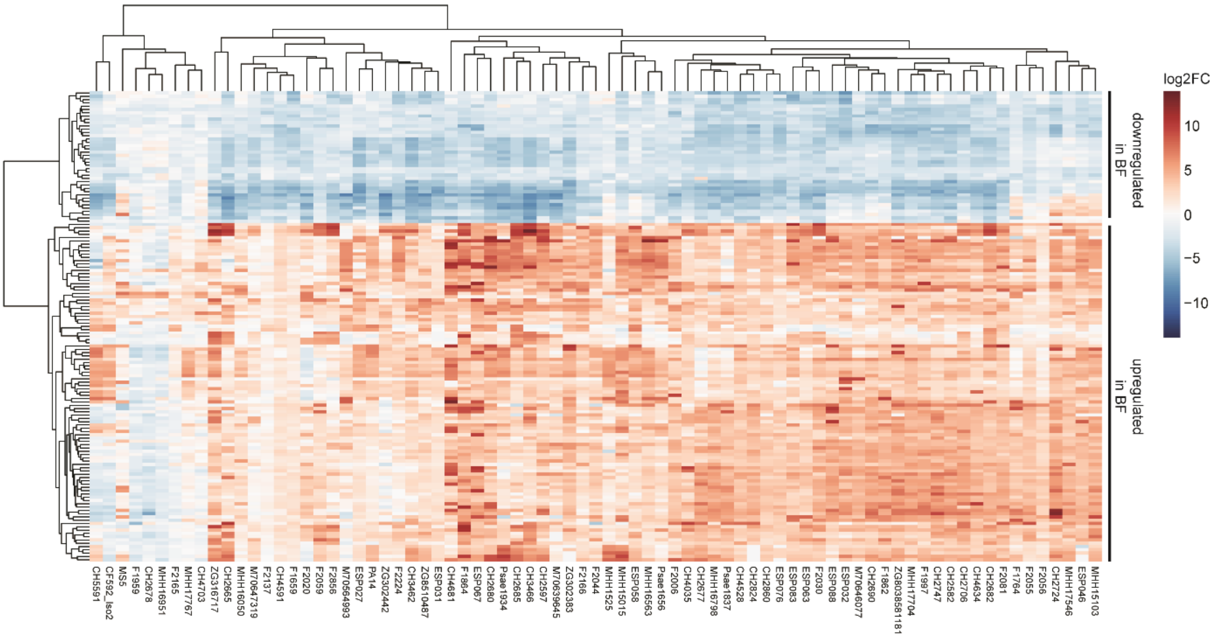

Supplementary Figure 3: Identified genes of the biofilm core transcriptome show a distinct expression pattern in an array of clinical isolates.

Individual expression values (log2-fold changes between biofilm [BF] and planktonic growth) of 143 genes belonging to the core biofilm transcriptome (upregulated in BF: 103 genes; downregulated in BF: 40 genes) are displayed for 77 strains in biofilm conditions compared to planktonic growth.

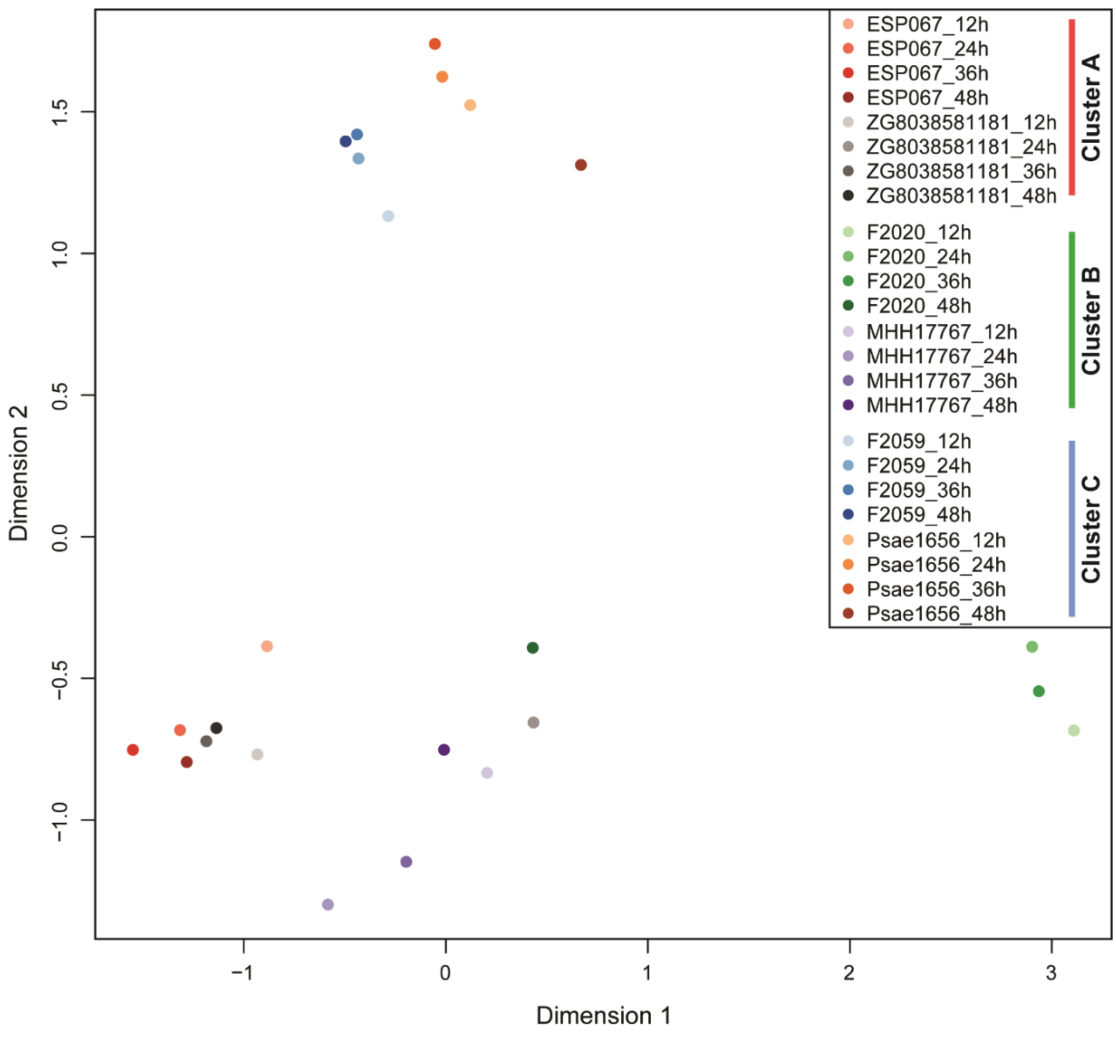

Supplementary Figure 4: Transcriptional profiles of biofilm samples over time.

The multidimensional scaling plot (MDS) of transcriptional profiles of six selected clinical isolates (two strains per each of the three biofilm clusters) reveals rather conserved profiles over time (12 h, 24 h, 36 h, and 48 h of biofilm growth), indicating that the maturation status of the biofilm has minor influence on transcriptional signatures under biofilm growth conditions. Each dot represents one clinical isolate under a certain biofilm condition.

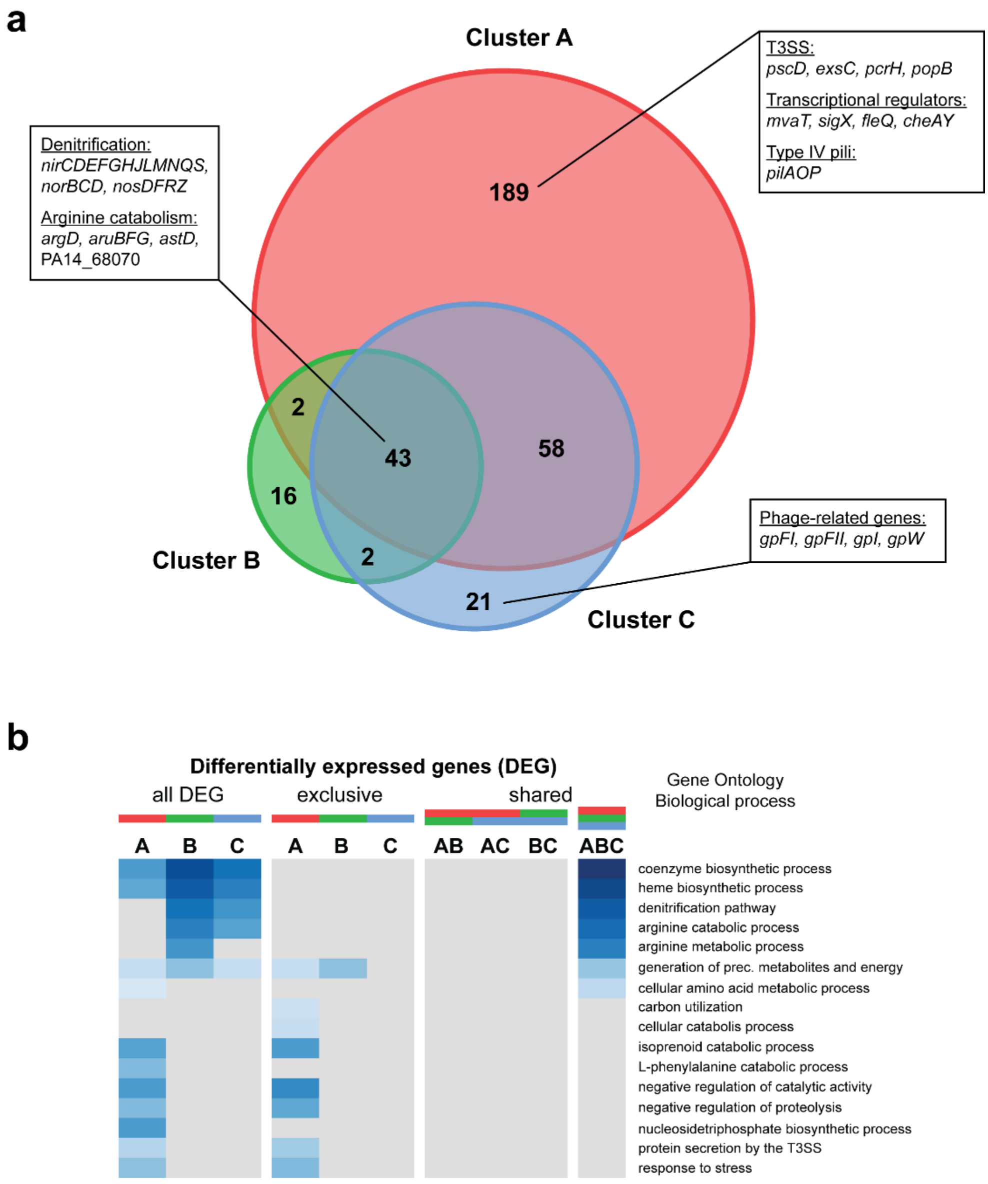

Supplementary Figure 5: Downregulated genes in biofilm growth in comparison to planktonic growth.

a. The Venn diagram depicts commonly downregulated genes (43 genes) among all three biofilm clusters as well as cluster-specific regulated genes (A: 189; B: 16; C: 21 genes). **b.** The GO term enrichment analysis of downregulated genes shows biological functions that are exclusively regulated in a certain biofilm cluster or shared by two or all three structural groups. The blue color gradient represents the value of the enrichment factor.

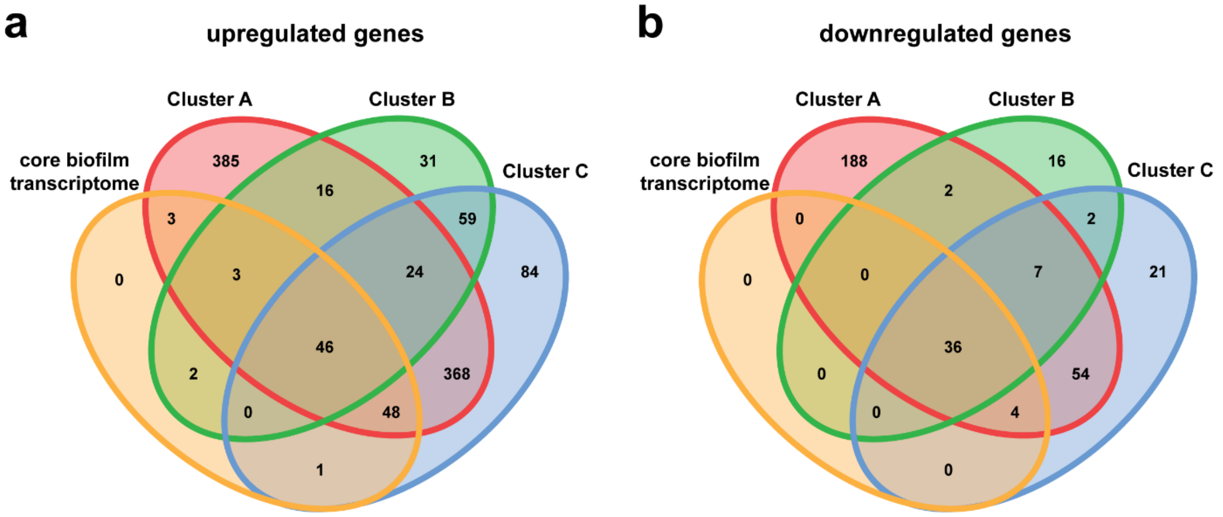

Supplementary Figure 6: Overlap of differentially expressed genes between the biofilm core and the three structurally distinct biofilm clusters.

a. Genes, which are specifically upregulated under biofilm growth conditions (threshold $\log2FC \geq 2$ with $FDR < 0.05$), share a large overlap between the core biofilm transcriptome (orange) and cluster A (red) and cluster C (blue), whereas in cluster B (green) only approximately half of the regulated genes are also included in the core biofilm transcriptome. **b.** Genes, which are specifically downregulated under biofilm growth conditions (threshold $\log2FC \leq -2$; $FDR < 0.05$), are largely shared between the core biofilm transcriptome and the three biofilm clusters.

Supplementary Table 1: Clinical isolates and the reference strain PA14 used for RNA-sequencing and phenotypic characterization

Strains used in this study are listed including a unique identifier ("Isolate name"), and available information about the geographic origin and sampling site. Clinical isolates that were assigned to one of the five identified biofilm clusters are labeled with the respective letter. The top 33 strains were used for extensive phenotypic characterization. CF – cystic fibrosis; nd – not determined.

	BF cluster	Isolate name	Clinic / geographic origin	Sampling site
Phenotypically characterized	A	CH2860	Charité Berlin	respiratory tract
	A	CH4528	Charité Berlin	respiratory tract
	A	ESP046	Palma de Mallorca, Spain[#]	nd/other
	A	ESP067	Palma de Mallorca, Spain[#]	nd/other
	A	ESP088	Palma de Mallorca, Spain[#]	nd/other
	A	F2030	University hospital Frankfurt	respiratory tract
	A	F2137	University hospital Frankfurt	wound
	A	MHH16798	Hannover Medical School	respiratory tract
	A	MHH17704	Hannover Medical School	nd/other
	A	ZG302383	Görlitz	nd/other
	A	ZG8038581181	Chemnitz	respiratory tract
	B	CH2682	Charité Berlin	rectal swab
	B	ESP027	Palma de Mallorca, Spain[#]	nd/other
	B	F1959	University hospital Frankfurt	respiratory tract, CF
	B	F2020	University hospital Frankfurt	wound
	B	F2165	University hospital Frankfurt	respiratory tract
	B	F2166	University hospital Frankfurt	respiratory tract
	B	F2224	University hospital Frankfurt	nd/other
	B	MHH16050	Hannover Medical School	nd/other
	B	MHH16563	Hannover Medical School	respiratory tract, CF
	B	MHH17767	Hannover Medical School	respiratory tract, CF
	C	CH2706	Charité Berlin	rectal swab
	C	CH4591	Charité Berlin	rectal swab
	C	CH4634	Charité Berlin	nd/other
	C	ESP063	Palma de Mallorca, Spain[#]	nd/other
	C	ESP083	Palma de Mallorca, Spain[#]	nd/other
	C	F1864	University hospital Frankfurt	nd/other
	C	F2006	University hospital Frankfurt	wound
	C	F2059	University hospital Frankfurt	wound
	C	F2856	University hospital Frankfurt	nd/other
	C	Psae1656	Munich*	respiratory tract
	C	ZG316717	Görlitz	ear
	C	ZG8510487	Leipzig	urinary tract
		PA14	Reference strain	burn wound
	C	CF592_Iso2	University hospital Essen	respiratory tract, CF
		CH2582	Charité Berlin	nd/other
		CH2597	Charité Berlin	wound

	CH2665	Charité Berlin	respiratory tract
	CH2677	Charité Berlin	urinary tract
	CH2678	Charité Berlin	respiratory tract, CF
	CH2680	Charité Berlin	nd/other
	CH2685	Charité Berlin	rectal swab
A	CH2690	Charité Berlin	rectal swab
A	CH2724	Charité Berlin	nd/other
	CH2747	Charité Berlin	respiratory tract
	CH2824	Charité Berlin	respiratory tract
	CH3462	Charité Berlin	rectal swab
	CH3466	Charité Berlin	sepsis
	CH4035	Charité Berlin	respiratory tract
B	CH4681	Charité Berlin	respiratory tract
	CH4703	Charité Berlin	nd/other
	CH5591	Charité Berlin	rectal swab
	ESP031	Palma de Mallorca, Spain[#]	nd/other
	ESP032	Palma de Mallorca, Spain[#]	nd/other
	ESP058	Palma de Mallorca, Spain[#]	nd/other
	ESP076	Palma de Mallorca, Spain[#]	nd/other
C	F1659	University hospital Frankfurt	respiratory tract
B	F1764	University hospital Frankfurt	respiratory tract
C	F1862	University hospital Frankfurt	respiratory tract
A	F1997	University hospital Frankfurt	rectal swab
	F2044	University hospital Frankfurt	respiratory tract
	F2055	University hospital Frankfurt	urinary tract
C	F2056	University hospital Frankfurt	wound
	F2081	University hospital Frankfurt	rectal swab
B	M70564993	University hospital Münster	respiratory tract
	M70639645	University hospital Münster	urinary tract
A	M70646077	University hospital Münster	rectal swab
B	M70647319	University hospital Münster	respiratory tract, CF
	MHH15015	Hannover Medical School	respiratory tract
	MHH15103	Hannover Medical School	wound
B	MHH1525	Hannover Medical School	respiratory tract, CF
	MHH16951	Hannover Medical School	respiratory tract, CF
	MHH17546	Hannover Medical School	respiratory tract, CF
	MS5	University hospital Münster	nd/other
	Psae1837	Gera*	nd/other
A	Psae1934	Bremen*	respiratory tract
	ZG302442	Görlitz	ear

[#] provided by a Spanish strain collection located at Son Espases University hospital in Palma de Mallorca, Spain
* provided by a strain collection located at University Hospital Freiburg

Supplementary Table 2: **Expression of *phz* genes**

Cluster-wise comparison of the expression of *phz* genes in biofilm (BF), or planktonic (PL) growth conditions. Significant changes (FDR < 0.05) are depicted in black; log2-fold changes (log2FC) above or below the threshold (±1.5) are displayed in bold.

PA14 locus tag	Gene	biofilm growth						planktonic growth					
		A vs. B		A vs. C		B vs. C		A vs. B		A vs. C		B vs. C	
		log2 FC	FDR	log2 FC	FDR	log2 FC	FDR	log2 FC	FDR	log2 FC	FDR	log2 FC	FDR
PA14_00640	*phzH*	1.0	0.114	0.9	0.210	-0.1	0.883	0.1	0.951	1.2	0.371	1.0	0.328
PA14_09400	*phzS*	0.2	0.882	0.1	0.955	-0.1	0.952	**4.0**	0.021	**4.2**	0.008	0.2	0.936
PA14_09410	*phzG1*	0.4	0.708	0.0	0.994	-0.4	0.768	3.1	0.061	**3.9**	0.010	0.7	0.714
PA14_09420	*phzF1*	0.1	0.964	-0.1	0.932	-0.2	0.909	3.3	0.056	**4.1**	0.009	0.7	0.717
PA14_09440	*phzE1*	0.4	0.715	0.3	0.801	-0.1	0.953	3.1	0.059	**4.0**	0.006	0.9	0.598
PA14_09450	*phzD1*	-0.5	0.689	0.2	0.907	0.6	0.663	**4.1**	0.021	**5.1**	0.002	1.0	0.624
PA14_09460	*phzC1*	0.2	0.863	0.2	0.907	-0.1	0.977	**4.5**	0.014	**5.3**	0.002	0.8	0.720
PA14_09470	*phzB1*	0.6	0.668	-0.6	0.689	-1.1	0.464	**4.0**	0.046	**5.3**	0.004	1.4	0.496
PA14_09480	*phzA1*	0.6	0.648	-0.3	0.802	-0.9	0.543	3.7	0.054	**5.2**	0.004	1.5	0.446
PA14_09490	*phzM*	0.3	0.737	-0.1	0.927	-0.4	0.727	2.3	0.055	**3.3**	0.002	1.0	0.433
PA14_39880	*phzG2*	0.4	0.689	0.0	0.994	-0.4	0.769	3.2	0.057	**3.8**	0.012	0.6	0.793
PA14_39890	*phzF2*	0.1	0.944	-0.1	0.934	-0.2	0.898	3.4	0.055	**4.1**	0.010	0.7	0.743
PA14_39910	*phzE2*	0.3	0.781	0.2	0.865	-0.1	0.956	3.1	0.058	**4.0**	0.007	0.9	0.632
PA14_39925	*phzD2*	-0.5	0.662	0.1	0.958	0.6	0.687	**3.9**	0.029	**4.6**	0.006	0.7	0.773
PA14_39945	*phzC2*	0.2	0.897	0.3	0.805	0.2	0.919	**4.0**	0.018	**4.9**	0.002	0.9	0.657
PA14_39960	*phzB2*	0.8	0.399	0.6	0.553	-0.2	0.897	**3.0**	0.034	**4.0**	0.002	1.0	0.505
PA14_39970	*phzA2*	0.8	0.437	0.1	0.918	-0.7	0.613	**3.3**	0.043	**4.7**	0.002	1.3	0.452

Supplementary Table 3: **Expression of T3SS-related genes**

Cluster-wise comparison of the expression of type III secretion system (T3SS)-related genes in biofilm (BF) or planktonic (PL) growth conditions. Significant changes (FDR < 0.05) are depicted in black; log2-fold changes (log2FC) above or below the threshold (±1.5) are displayed in bold.

PA14 locus tag	Gene	biofilm growth						planktonic growth					
		A vs. B		A vs. C		B vs. C		A vs. B		A vs. C		B vs. C	
		log2 FC	FDR	log2 FC	FDR	log2 FC	FDR	log2 FC	FDR	log2 FC	FDR	log2 FC	FDR
PA14_00560	*exoT*	1.0	0.182	**-2.0**	0.022	**-3.0**	0.003	0.8	0.697	-1.7	0.187	**-2.5**	0.033
PA14_14330	-	1.2	0.129	-0.4	0.659	-1.6	0.102	1.1	0.523	-0.8	0.659	-2.0	0.119
PA14_36345	*exoY*	2.2	0.051	-1.0	0.404	**-3.3**	0.029	0.0	0.997	-2.2	0.294	-2.2	0.232
PA14_42250	*pscL*	1.4	0.008	-0.4	0.470	**-1.9**	0.006	0.6	0.686	-0.7	0.579	-1.3	0.148
PA14_42260	*pscK*	1.5	0.006	-0.4	0.483	**-1.9**	0.005	0.6	0.646	-0.6	0.659	-1.2	0.164
PA14_42270	*pscJ*	**1.9**	0.001	-0.3	0.671	**-2.2**	0.002	0.5	0.734	-0.9	0.427	-1.4	0.107
PA14_42280	*pscI*	0.8	0.177	-1.2	0.090	**-2.0**	0.012	0.7	0.695	-0.8	0.598	-1.4	0.161
PA14_42290	*pscH*	1.3	0.053	-0.8	0.271	**-2.0**	0.014	1.1	0.438	-0.5	0.754	-1.6	0.136
PA14_42300	*pscG*	0.5	0.535	**-1.7**	0.043	**-2.2**	0.021	1.0	0.596	-0.9	0.588	-1.9	0.114
PA14_42310	*pscF*	1.1	0.164	-1.1	0.187	**-2.1**	0.027	1.2	0.486	-1.1	0.505	-2.3	0.056
PA14_42320	*pscE*	-0.7	0.454	**-3.5**	0.004	**-2.8**	0.021	1.5	0.420	-0.5	0.846	-2.1	0.174
PA14_42340	*pscD*	0.7	0.438	**-2.0**	0.043	**-2.7**	0.017	1.5	0.391	-0.4	0.870	-1.9	0.171
PA14_42350	*pscC*	1.4	0.039	-0.9	0.216	**-2.3**	0.009	0.7	0.700	-1.0	0.504	-1.7	0.125
PA14_42360	*pscB*	1.4	0.051	-0.8	0.313	**-2.2**	0.018	0.9	0.646	-0.8	0.655	-1.6	0.167
PA14_42380	*exsD*	0.1	0.853	**-2.3**	0.005	**-2.5**	0.004	0.9	0.563	-0.7	0.667	-1.6	0.138
PA14_42390	*exsA*	0.3	0.698	-1.0	0.133	-1.3	0.096	0.8	0.602	-0.6	0.698	-1.4	0.167
PA14_42400	*exsB*	0.3	0.719	**-2.1**	0.016	**-2.4**	0.013	0.3	0.882	-0.9	0.606	-1.2	0.296
PA14_42410	-	-1.1	0.203	**-3.6**	0.002	**-2.5**	0.022	0.4	0.891	-1.0	0.608	-1.4	0.309
PA14_42430	*exsC*	-0.9	0.218	**-3.4**	0.002	**-2.5**	0.009	0.7	0.714	-0.7	0.688	-1.4	0.226
PA14_42440	*popD*	0.6	0.513	**-3.6**	0.004	**-4.2**	0.002	1.3	0.591	-1.6	0.403	-2.9	0.058
PA14_42450	*popB*	0.3	0.781	**-3.7**	0.005	**-4.0**	0.004	1.3	0.612	-1.8	0.375	-3.1	0.058
PA14_42460	*pcrH*	0.0	0.968	**-3.7**	0.004	**-3.7**	0.005	1.1	0.675	-1.0	0.673	-2.1	0.194
PA14_42470	*pcrV*	1.0	0.228	**-2.4**	0.017	**-3.4**	0.003	1.0	0.663	-1.4	0.429	-2.4	0.081
PA14_42480	*pcrG*	0.8	0.397	**-3.7**	0.003	**-4.5**	0.002	1.4	0.535	-1.1	0.606	-2.5	0.107
PA14_42490	*pcrR*	0.8	0.195	-1.5	0.043	**-2.3**	0.006	0.5	0.775	-0.3	0.860	-0.9	0.400
PA14_42500	*pcrD*	1.4	0.017	-0.4	0.500	**-1.8**	0.013	0.4	0.821	-0.8	0.503	-1.2	0.193
PA14_42510	*pcr4*	**1.6**	0.040	-0.3	0.725	**-1.9**	0.050	0.0	0.994	-0.9	0.640	-0.8	0.516
PA14_42520	*pcr3*	0.8	0.327	-1.3	0.157	**-2.1**	0.047	0.4	0.881	-1.1	0.540	-1.5	0.252
PA14_42530	*pcr2*	1.3	0.116	-1.3	0.150	**-2.5**	0.016	0.2	0.944	-0.9	0.619	-1.1	0.383
PA14_42540	*pcr1*	**1.6**	0.039	-0.6	0.508	**-2.2**	0.028	0.1	0.979	-1.7	0.196	-1.8	0.170
PA14_42550	*popN*	0.5	0.563	-1.2	0.186	-1.7	0.102	0.4	0.864	-1.0	0.610	-1.5	0.288
PA14_42570	*pscN*	1.6	0.051	-0.7	0.460	**-2.3**	0.030	0.6	0.800	-1.4	0.383	-2.0	0.136
PA14_42580	*pscO*	1.4	0.105	-1.3	0.157	**-2.7**	0.016	0.4	0.893	-1.1	0.567	-1.5	0.283
PA14_42600	*pscP*	**2.8**	0.001	-0.1	0.930	**-2.9**	0.004	0.8	0.695	-0.6	0.770	-1.4	0.253
PA14_42610	*pscQ*	1.5	0.062	-0.5	0.622	-2.0	0.053	1.0	0.619	-0.9	0.654	-1.9	0.154
PA14_42620	*pscR*	**2.1**	0.004	0.3	0.707	**-1.8**	0.045	0.8	0.674	-0.6	0.737	-1.4	0.220
PA14_42630	*pscS*	**2.3**	0.001	0.4	0.633	**-1.9**	0.021	0.8	0.626	-0.7	0.681	-1.5	0.167
PA14_42640	*pscT*	**2.0**	0.004	0.4	0.597	-1.6	0.055	0.1	0.985	-0.5	0.770	-0.6	0.637
PA14_42660	*pscU*	**2.8**	0.000	0.9	0.165	**-1.9**	0.013	0.2	0.929	-0.5	0.749	-0.7	0.511
PA14_51520	*spcU*	0.0	0.994	-4.4	0.090	-4.4	0.133	0.3	0.975	-0.7	0.914	-1.0	0.819
PA14_51530	*exoU*	1.0	0.678	-3.9	0.135	-4.9	0.119	1.8	0.782	-0.8	0.908	-2.6	0.504
PA14_00560	*exoT*	1.0	0.182	**-2.0**	0.022	**-3.0**	0.003	0.8	0.697	-1.7	0.187	**-2.5**	0.033
PA14_14330	-	1.2	0.129	-0.4	0.659	-1.6	0.102	1.1	0.523	-0.8	0.659	-2.0	0.119
PA14_36345	*exoY*	2.2	0.051	-1.0	0.404	**-3.3**	0.029	0.0	0.997	-2.2	0.294	-2.2	0.232
PA14_42250	*pscL*	1.4	0.008	-0.4	0.470	**-1.9**	0.006	0.6	0.686	-0.7	0.579	-1.3	0.148
PA14_42260	*pscK*	1.5	0.006	-0.4	0.483	**-1.9**	0.005	0.6	0.646	-0.6	0.659	-1.2	0.164
PA14_42270	*pscJ*	**1.9**	0.001	-0.3	0.671	**-2.2**	0.002	0.5	0.734	-0.9	0.427	-1.4	0.107
PA14_42280	*pscI*	0.8	0.177	-1.2	0.090	**-2.0**	0.012	0.7	0.695	-0.8	0.598	-1.4	0.161
PA14_42290	*pscH*	1.3	0.053	-0.8	0.271	**-2.0**	0.014	1.1	0.438	-0.5	0.754	-1.6	0.136
PA14_42300	*pscG*	0.5	0.535	**-1.7**	0.043	**-2.2**	0.021	1.0	0.596	-0.9	0.588	-1.9	0.114
PA14_42310	*pscF*	1.1	0.164	-1.1	0.187	**-2.1**	0.027	1.2	0.486	-1.1	0.505	-2.3	0.056

Supplementary Table 4: Phenotypic characterization of 33 selected clinical isolates

Colony size: 0 – SCV to 3 – large; Margin: 0 – no spreading to 2 – strong spreading of the colony; Surface: 0 – rough, 1 – smooth and shiny; metallic sheen: 0 – none to 2 – clearly visible throughout the bacterial lawn; Hemolysis: 0 – none to 2 – enhanced; growth iron limitation: 1 – growth, 2 – no growth.

Isolate	Biofilm cluster	PA14 - PAO1 like	Colony size	Margin	Surface	Metallic sheen	Hemolysis	Growth under iron-limitation	Biofilm biomass [OD_{540}]	c-di-GMP [pmol/mg protein]	Virulence in *G. mellonella* [% killing]	Cytotoxicity [%]	Pyocyanin [µg/ml]	rel. Elastase secretion 24 h planktonic	rel. Elastase secretion 48 h biofilm	rel. Protease production	Swimming zone [cm²]	Twitching area [cm²]	Swarming area [cm²]	H_2O_2 diameter inhibition zone [mm]
CH2860	A	PAO1	3	1	1	0	2	1	0.36	1.70	95%	35%	5.46	1.00	0.85	0.29	0.98	4.51	0.46	16.56
CH4528	A	PAO1	2	1	0	2	1	1	0.76	3.37	100%	31%	7.22	0.49	0.53	0.27	0.40	4.47	0.00	19.33
ESP046	A	PAO1	2	2	1	0	2	1	0.42	2.39	100%	34%	2.95	0.88	0.77	0.34	0.51	2.90	2.36	15.67
ESP067	A	PAO1	1	2	0	2	2	1	0.49	2.82	95%	45%	3.07	0.84	1.00	0.47	1.90	2.25	6.21	16.33
ESP088	A	PAO1	2	1	1	0	2	1	0.56	2.79	95%	34%	0.00	0.06	0.12	0.12	0.41	2.12	0.00	17.67
F2030	A	PA14	2	1	0	1	2	1	1.92	5.85	100%	0%	0.09	0.42	0.67	0.80	0.58	0.53	0.00	19.17
F2137	A	PAO1	2	1	0	2	1	1	0.17	9.19	80%	63%	0.60	0.02	0.79	0.00	0.28	2.08	0.00	17.50
MHH16798	A	PAO1	2	1	1	0	2	1	0.47	3.87	80%	36%	1.03	0.84	0.98	0.27	0.69	1.71	0.31	17.17
MHH17704	A	PAO1	#NA	#NA	#NA	#NA	#NA	1	0.95	3.14	95%	22%	0.07	0.54	0.67	0.32	1.02	4.66	0.28	18.50
ZG302383	A	PAO1	2	1	1	0	2	1	0.81	5.90	100%	60%	2.09	0.70	0.79	0.38	1.01	1.49	2.66	19.61
ZG8038581181	A	PAO1	2	1	1	0	2	1	0.59	2.23	90%	31%	1.29	0.97	0.97	0.66	0.87	1.42	16.01	17.67
CH2682	B	PAO1	3	0	1	0	2	0	0.54	4.68	5%	7%	0.05	#NA	#NA	0.20	0.00	1.11	0.36	17.17
ESP027	B	PAO1	0	0	0	1	0	0	0.73	2.91	0%	3%	0.21	0.27	0.20	0.00	0.68	0.30	0.00	18.00
F1959	B	PAO1	0	0	0	0	0	0	0.01	93.66	0%	13%	0.07	0.68	0.00	0.26	0.00	0.00	0.00	8.61
F2020	B	PA14	3	0	0	1	1	1	0.09	7.27	5%	0%	0.08	0.01	0.03	0.05	0.69	0.15	0.00	13.33
F2165	B	PAO1	2	0	0	2	1	1	0.13	11.93	0%	5%	0.37	0.03	0.54	0.00	0.19	1.62	0.00	17.17
F2166	B	PAO1	3	0	0	2	1	1	0.12	13.36	0%	5%	1.77	0.03	0.54	0.00	0.24	1.11	0.00	16.83
F2224	B	PAO1	2	0	1	0	1	1	1.01	22.11	5%	27%	0.15	0.05	0.11	0.19	0.29	0.70	0.00	16.17
MHH16050	B	PAO1	1	0	0	1	1	1	0.01	2.75	40%	1%	0.14	0.03	0.00	0.00	0.00	0.57	0.00	18.33
MHH16563	B	PA14	1	1	1	0	1	1	1.70	63.67	5%	27%	0.03	0.15	0.35	0.47	0.27	1.94	0.00	17.00
MHH17767	B	PAO1	0	0	0	1	0	1	0.58	2.42	0%	41%	1.73	0.06	0.69	0.00	0.10	0.37	0.00	10.67
CH2706	C	PAO1	2	1	0	1	1	1	1.65	4.00	100%	50%	1.27	0.36	0.06	0.15	0.17	2.92	0.21	14.33
CH4591	C	PAO1	2	0	0	1	1	1	0.72	3.63	100%	47%	0.40	0.70	0.63	0.42	1.52	2.18	0.00	14.00
CH4634	C	PA14	2	0	1	0	1	1	0.70	2.96	100%	25%	1.58	0.74	0.80	0.72	0.56	4.01	9.91	17.83
ESP063	C	PAO1	2	1	0	2	1	1	0.30	1.76	100%	53%	0.00	0.01	0.06	0.06	0.24	3.40	0.00	19.67
ESP083	C	PAO1	2	1	0	2	1	1	0.34	4.01	100%	54%	0.03	0.01	0.03	0.00	0.40	2.85	0.00	17.67
F1864	C	PA14	2	1	0	2	1	1	0.80	2.77	100%	51%	0.02	0.01	0.13	0.10	0.15	2.37	0.00	19.25
F2006	C	PAO1	1	0	0	1	1	1	1.43	12.65	15%	52%	2.21	0.03	0.40	0.00	0.32	1.03	0.20	20.92
F2059	C	PAO1	2	1	0	2	1	1	1.18	7.17	100%	50%	0.11	0.01	0.05	0.16	0.85	0.00	0.00	18.42
F2856	C	PAO1	1	1	0	2	1	1	0.62	3.53	35%	43%	0.13	0.01	0.02	0.24	0.90	3.16	0.00	18.00
Psae1656	C	PA14	2	1	0	2	1	1	2.76	6.37	70%	44%	0.08	0.01	0.02	0.00	0.00	1.87	0.00	17.75
ZG316717	C	PA14	0	0	0	1	1	0	2.42	3.51	95%	47%	0.12	0.01	0.05	0.00	0.35	0.61	0.00	19.83
ZG8510487	C	PAO1	1	1	0	2	1	1	2.17	2.93	100%	32%	1.01	0.28	0.98	0.31	0.00	0.12	0.00	18.17

Description of additional electronic Supplementary Data

Supplementary Data 1:

Differential gene expression analysis under biofilm (BF) versus planktonic (PL) growth conditions for 77 diverse clinical isolates (all 77 isolates) and for three major structurally distinct biofilm clusters (cluster A, cluster B, cluster C).

Supplementary Data 2:

Cluster-wise comparison of gene expression for transcriptional profiles in biofilm (BF) or planktonic (PL) growth conditions.
